# Leaf size of woody dicots predicts ecosystem primary productivity

**DOI:** 10.1111/ele.13503

**Published:** 2020-04-06

**Authors:** Yaoqi Li, Peter B. Reich, Bernhard Schmid, Nawal Shrestha, Xiao Feng, Tong Lyu, Brian S. Maitner, Xiaoting Xu, Yichao Li, Dongting Zou, Zheng‐Hong Tan, Xiangyan Su, Zhiyao Tang, Qinghua Guo, Xiaojuan Feng, Brian J. Enquist, Zhiheng Wang

**Affiliations:** ^1^ Institute of Ecology and Key Laboratory for Earth Surface Processes of the Ministry of Education College of Urban and Environmental Sciences Peking University Beijing 100871 China; ^2^ Department of Forest Resources University of Minnesota St. Paul MN 55108 USA; ^3^ Hawkesbury Institute for the Environment Western Sydney University Penrith NSW 2751 Australia; ^4^ Department of Geography Remote Sensing Laboratories University of Zurich Winterthurerstrasse 190 8057 Zurich Switzerland; ^5^ Institute of Innovation Ecology Lanzhou University Lanzhou 730000 China; ^6^ Institute of the Environment University of Arizona Tucson Arizona 85721 USA; ^7^ Department of Ecology and Evolutionary Biology University of Arizona Tucson AZ 85721 USA; ^8^ Key Laboratory of Bio‐Resource and Eco‐Environment of Ministry of Education College of Life Sciences Sichuan University Chengdu 610065 Sichuan China; ^9^ College of Environment and Ecology Hainan University Haikou Hainan 570228 China; ^10^ State Key Laboratory of Vegetation and Environmental Change Institute of Botany Chinese Academy of Sciences Beijing 100093 China; ^11^ The Santa Fe Institute Santa Fe NM 87501 USA

**Keywords:** Annual evapotranspiration, China, community mean leaf size, large‐scale eco‐evolutionary patterns, leaf area index, North America, palaeo‐primary productivity, plant functional traits

## Abstract

A key challenge in ecology is to understand the relationships between organismal traits and ecosystem processes. Here, with a novel dataset of leaf length and width for 10 480 woody dicots in China and 2374 in North America, we show that the variation in community mean leaf size is highly correlated with the variation in climate and ecosystem primary productivity, independent of plant life form. These relationships likely reflect how natural selection modifies leaf size across varying climates in conjunction with how climate influences canopy total leaf area. We find that the leaf size‒primary productivity functions based on the Chinese dataset can predict productivity in North America and vice‐versa. In addition to advancing understanding of the relationship between a climate‐driven trait and ecosystem functioning, our findings suggest that leaf size can also be a promising tool in palaeoecology for scaling from fossil leaves to palaeo‐primary productivity of woody ecosystems.

## Graphical abstract

Based on leaf size and distribution data for 10 480 woody dicots in China and 2374 in North America, we found that leaf size of woody dicots well predicts ecosystem primary productivity both in China and North America, and its independent and direct effects on primary productivity is higher than (or in a few cases comparable to) those of climate, soil characteristics and leaf area index. Our results suggest that leaf size provides a promising surrogate to reconstruct palaeo‐primary productivity of terrestrial woody ecosystems based on fossil leaf deposits.

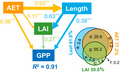

## Introduction

Plant traits at species level have been determined by selection processes that operate on the fitness of individuals (Ackerly *et al.* 2000; Reich *et al.* 2003; Donovan *et al.* 2011; Zanne *et al.* 2014). These traits, nonetheless, influence processes at the community and ecosystem scale (Reich *et al.* 1992; Reich 2014; Reichstein *et al.* 2014), and both the variations in traits and ecosystem processes are also independently influenced by climatic variations (Nemani *et al.* 2003; Zhao & Running 2010; Wright *et al.* 2017). Despite considerable advances in the field (Lavorel & Garnier 2002; Reichstein *et al.* 2014), whether and how plant traits, such as leaf size, are related to ecosystem processes remains an important knowledge gap in terrestrial ecology.

Ecosystem primary productivity, typically measured as the cumulative gross photosynthesis or net biomass production at ecosystem level, is a key ecosystem function that plays a central role in the global carbon cycle and mitigation of climate change (Lieth 1975; Nemani *et al.* 2003; Zhao & Running 2010). Additionally, assessing current and reconstructing past primary productivity is essential to understanding the long‐term evolution of ecosystems and their adaptation to climatic variation (Beaufort *et al.* 1997; Schubert *et al.* 1998). Better understanding of links between traits and productivity is a prime objective of recent research (Michaletz *et al.* 2014; Reichstein *et al.* 2014; Madani *et al.* 2018). One such challenge in trait‐based ecology is how to best predict variation in ecosystem primary productivity from the traits of individuals (Sack & Scoffoni 2013; Violle *et al.* 2014). Previous studies show that leaf functional traits are closely related to leaf photosynthesis and plant growth rates (Reich *et al.* 1997; Wright *et al.* 2004; Enquist *et al.* 2007). These leaf traits can be scaled up from leaves to ecosystem functioning (Reich *et al.* 1997; Garnier *et al.* 2004; Reich 2012; Madani *et al.* 2018), thereby providing a potential tool to estimate terrestrial primary productivity, as well as enhance our understanding of trait–ecosystem functioning relationships.

Leaf size is a trait that represents the trade‐off between carbon assimilation and water‐use efficiency, and is crucial to leaf thermoregulation across different climates (Michaletz *et al.* 2016; Fauset *et al.* 2018). It influences leaf energy balance through radiative warming and transpirational cooling via leaf boundary layers (Schuepp 1993; Michaletz *et al.* 2016). Studies show that leaf size significantly varies with precipitation and temperature (Parkhurst & Loucks 1972; Moles *et al.* 2014; Wright *et al.* 2017), although the relative importance of these environmental variables across regions and communities with different species compositions is yet to be fully understood.

Variation in leaf size across different climates may reflect physiological, biomechanical and hydraulic adaptations of plants to avoid night‐time frost (Wright *et al.* 2017), to maximise water‐use efficiency (Fauset *et al.* 2018), and to maintain day‐time optimal (or maximum) photosynthesis (Michaletz *et al.* 2016). In drylands and cold regions, small leaves can avoid day‐time overheating and night‐time frost and, thus, are able to maintain photosynthetic functions (Wright *et al.* 2017). With increasing water and energy availability, large leaves with thick boundary layers can maintain high water and CO_2_ exchange rates, day‐time leaf temperature and hence high photosynthetic rates (Givnish 1978; Michaletz *et al.* 2016; Wright *et al.* 2017). Moreover, warmer, wetter and less seasonal environments are also associated with greater leaf area index (LAI), higher light interception and carbon uptake and, hence, increased annual primary productivity (Gower *et al.* 1999; Reich 2012). These findings suggest that geographical variation in leaf size may be closely related to geographical variation in terrestrial primary productivity, both as a potential driver of such processes and via co‐variation with other drivers.

As effects of leaf traits on net photosynthesis can be scaled up from leaves to ecosystem functioning (Reich *et al.* 1997; Garnier *et al.* 2004), we ask whether climate may influence ecosystem primary productivity via its influence on leaf size. Exploring the co‐variation between climate, leaf size and primary productivity across ecosystems and further partitioning the contributions of climate and leaf size to the variation in primary productivity would help to test this hypothesis that climatic effects on primary productivity are, at least in part, mediated by leaf size. Moreover, recent studies indicate that relationships between leaf traits and climate vary among plants with different life forms and evolutionary history (Jordan 2011; Li *et al.* 2016). However, whether life form and evolutionary history modifies potential relationships of leaf size with climate and primary productivity remains to be evaluated.

Reconstructing palaeo‐primary productivity is critical to understanding the dynamics of ecosystem functioning in response to historical climate change. Previous reconstructions of palaeo‐primary productivity are mainly limited to aquatic ecosystems, employing methods including biomarkers and aquatic microfossil assemblages (Beaufort *et al.* 1997; Schubert *et al.* 1998). However, the reconstruction of terrestrial primary productivity is limited by the lack of appropriate surrogates. The total leaf area of canopy per unit ground area (i.e. LAI), is a surrogate of canopy capacity for light interception and carbon uptake and has been used to model current forest productivity (Gower *et al.* 1999; Reich 2012). However, LAI is not suitable for reconstructing palaeo‐primary productivity due to difficulties in estimating palaeo‐LAI (Dunn *et al.* 2015). Therefore, establishing relationships between leaf size and primary productivity could provide a new method for reconstructing terrestrial palaeo‐primary productivity from well‐preserved leaf fossils.

Here, we use a dataset of leaf sizes and geographical distributions of 10 480 woody dicot species in China and 2374 in North America to first assess whether there is a relationship between community mean leaf size and climate, and if so, whether it is modified by plant life form and evolutionary age. Second, we evaluate the performance of community mean leaf size as a predictor of ecosystem primary productivity, both in combination and comparison with climate, soil and LAI. Third, we build transfer functions between leaf size and primary productivity based on the Chinese dataset and evaluate the generality of these functions by applying them to the North American dataset, and vice versa.

## MATERIALS AND METHODS

### Leaf size, life forms and distributions of woody dicots in China

Our study focused on woody dicots. We compiled the leaf length and width ranges, along with life forms (trees, shrubs and lianas; deciduous and evergreen) for each species mainly from the *Flora of China* (http://www.efloras.org/flora_page.aspx?flora_id=2, accessed January 2014). Our database totally contained 10 480 Chinese woody dicots, with 9855 species (*c*. 94%) having leaf length and 9695 (*c*. 93%) having leaf width data. Previous studies multiplied leaf length by leaf width with a correction factor of 2/3 or 3/4 to account for the difference between rectangular and elliptical shape (Wilf *et al.* 1998; Cristofori *et al.* 2007; Rouphael *et al.* 2010). We calculated the 2/3 × leaf length × leaf width product (leaf length‐width product hereafter) as a proxy measure of leaf area. Species distribution maps were extracted from the *Atlas of Woody Plants in China: Distribution and Climate* (Fang *et al.* 2011) and transformed into species presence/absence data per grid cell of 50 × 50 km (see Data supplement for details).

### Leaf size and distributions of woody dicots in North America

Leaf size measures for all species in North America were mainly compiled from the *Flora of North America* (*FNA*, http://efloras.org/flora_page.aspx?flora_id=1, accessed June 2017). Species distributions were compiled from two different sources: a) occurrences and range maps from the *Botanical Information and Ecology Network* (BIEN) (accessed January 2018; see Appendix S5); b) political state‐/province‐level distributions from *FNA* and *USDA* plant databases (https://plants.usda.gov/java/ *,* accessed June 2017). Ultimately, 2374 dicot species were included (see Data supplement for details).

### Phylogenies of angiosperms

We obtained two dated family‐ and one genus‐level phylogenies from recent publications (Zanne *et al.* 2014; Magallon *et al.* 2015; Lu *et al.* 2018). We adjusted family names according to the *Angiosperm Phylogeny Website* (http://www.mobot.org/MOBOT/research/APweb/, accessed May 2016), and then extracted family and genus ages from each of the phylogenies separately.

### Environmental data

Spatial variations in leaf traits have been found to covary with climate (Wiemann *et al.* 1998; Wright *et al.* 2017). Here we used mean annual temperature (MAT), mean temperature of warmest quarter (MTWQ) and mean temperature of coldest quarter (MTCQ) to evaluate effects of temperature, mean annual precipitation (MAP), precipitation of wettest quarter (MPWQ) and aridity index (AI) to evaluate effects of precipitation and annual actual evapotranspiration (AET) to evaluate joint effect of available water and energy. AET includes estimated plant transpiration along with soil evaporation, is mainly driven by radiative energy and precipitation, and has been found to be strongly correlated with ecosystem primary productivity (Garbulsky *et al.* 2010; Yuan *et al.* 2010; Zhang *et al.* 2017). We also included mean annual solar radiation (SRAD) that affects leaf photosynthesis and transpiration. Climate data were obtained from the *WorldClim* database (http://www.worldclim.org/). Each environmental layer was resampled into the same resolution as those of species distributions in China and North America (see Data supplement for details).

Data on soil pH, soil organic carbon content and soil cation exchange capacity in the top 30‐cm soil layer were obtained from the *SoilGrids* (https://files.isric.org/soilgrids/data/, accessed by January 2019). We then conducted principal component analysis (PCA) on the three soil variables and extracted the first principal component which contains > 70% of edaphic variation (see Data supplement for details).

To test the applicability of community mean leaf size as a predictor for ecosystem primary productivity, we first obtained annual gross primary productivity (GPP) and net primary productivity (NPP) from 2000 to 2015 at 1 × 1 km resolution from the *Numerical Terradynamic Simulation Group* (http://www.ntsg.umt.edu/project/modis/mod17.php, accessed April 2017). This dataset was derived from a widely used Moderate Resolution Imaging Spectroradiometer (MODIS) product, and was calculated using the C5 MOD17 algorithm with data validation from flux towers (Zhao *et al.* 2005; Zhao & Running 2010). For comparison, we obtained a flux‐based GPP dataset (Yao *et al.* 2018) from 1982 to 2015 estimated with the Model Tree Ensemble algorithm at 0.1° resolution and an improved NPP dataset (Feng *et al.* 2019) from 1982 to 2015 estimated with the Carnegie‐Ames‐Stanford Approach (CASA) at 8‐km resolution. We also obtained biomass‐estimated NPP data for 1099 forest stands in China from a recent publication (Michaletz *et al.* 2014).

We resampled all annual GPP and NPP data to the spatial resolutions of species distributions in China and North America and calculated the mean values per grid cell for the entire period. The scaling up of productivity data to the resolution of leaf size estimates might induce uncertainties in leaf size–primary productivity relationships given the diversity of vegetation types within each grid cell. We compared GPP and NPP from different sources to explore their relationships with leaf size (Appendix S4). Furthermore, to evaluate how inter‐annual variations in GPP and NPP may influence leaf size–primary productivity relationships, we also calculated mean GPP and NPP of the highest 2, 4 and 8 years out of the 16 years for each grid cell and those of the lowest 2, 4 and 8 years based on the MODIS‐derived data.

To compare the effects of leaf size and LAI on primary productivity, we obtained LAI data from *GLOBMAP LAI* (http://www.modis.cn/globalLAI/GLOBMAPLAI_Version2/, accessed June 2017) with time resolutions of half‐months (1981–1999) or 8 days (2000–2015) generated from MODIS and Advanced Very High Resolution Radiometer data (Liu *et al.* 2012). We estimated the mean LAI in July per grid cell during the 35‐year period.

## Data analyses

### Geographical patterns of leaf size

For Chinese woody dicots, we calculated the community mean leaf length per grid cell using the median leaf length per species. Due to lack of abundance data at our spatial scale, leaf length was not weighted by species abundance but based on the presence/absence data. This approach may affect the relationships of leaf size with climate (Vile *et al.* 2006; Griffin‐Nolan *et al.* 2018), but its advantage is that the transfer functions derived from mean leaf size are more applicable for reconstruction of palaeo‐primary productivity because fossil depositions rarely contain information on species abundances. Similarly, we calculated the mean leaf width and mean leaf length‐width product per grid cell. We found strong correlations between these three measures (i.e. leaf length, leaf width and length‐width product; Appendix S1). We therefore use ‘leaf size’ to refer to the three measures in a generic way, except when we discuss results for specific metrics. Community mean leaf sizes were calculated separately for different life forms to evaluate the influence of life form on leaf size–environment relationships. We repeated all analyses using maximum leaf size per species to calculate community means and obtained very similar results (see Table S1.1). We therefore only present the results using community means based on median values per species in the main text.

For North America, we estimated mean leaf size per grid cell or per state/province, depending on the resolutions of two independent data sources, *BIEN* and *FNA* separately. Following previous studies (Wolfe 1993; Li *et al.* 2016), grid cells in China and North America with less than 20 species were removed from the following regressions (see Table S1.4) to eliminate potential influence of low species richness on relationships of leaf size with other variables.

### Phylogenetic signals and evolutionary age patterns

We calculated mean leaf size per family and per genus by averaging values of all species within each family and each genus in China, and estimated Blomberg’s *K* to evaluate the phylogenetic signals of leaf size using the R package “*phytools*” (Revell 2012). Spatial patterns of clade age have been used as a proxy for evolutionary history to test the effects of niche conservatism on forest diversity patterns and to explore hotspots of floral divergence (Hawkins *et al.* 2014; Lu *et al.* 2018). We therefore calculated mean clade (i.e. family and genus) age of all species appearing in each grid cell using the three phylogenies separately to test whether evolutionary age influenced leaf size–environment relationships.

### Relationships between leaf size and climate

Previous studies used linear models to fit the response of leaf traits to climate (Wolfe 1979; Wilf *et al.* 1998). Recently, nonlinear models were recommended to link traits with ecosystem properties (Reichstein *et al.* 2014). Here, we tried several regressions to evaluate relationships between leaf size and climate, including simple linear models (y = *a* +*b* +*x* and sqrt(*y*) = *a* +*b* +*x*, to linearise leaf size–climate relationships and normalise residual distributions), exponential models (y = *a*×*b^x^*), power models (y = *a* ×*x^b^*) and logistic models ($y\;=\;\frac{K}{1+e^{\alpha -rx}}$, x<K), where *x* and *y* are climate variables and leaf size, respectively, and *a*, *b*, K, *α* and *r* are regression coefficients. Considering the trade‐off between generality and precision, we finally chose the simple linear models and logistic models with the best performance. Modified *t*‐tests (Dutilleul *et al.* 1993) were used to assess the significance of regressions to avoid potential inflation of model significance due to spatial autocorrelation.

We conducted PCA and partial regression analyses to compare the relative effects of temperature and water availability on leaf size, and conducted hierarchical partitioning analyses (HPAs) using the R package ‘*hier.part*’ to compare effects of different variables.

### Relationships between leaf size and ecosystem primary productivity

We explored relationships of primary productivity (i.e. GPP and NPP) with leaf size using linear (y=a+b×x and sqrt(*y*) =* a* +*b* +*x*) and nonlinear ($y\;=\;\frac{1}{r}\left(\alpha -\ln\left(\frac{K}{x}-1\right)\right)$, x<K) regressions, in which *x* and *y* represent leaf size and primary productivity, respectively, and *a*, *b*, *α*, *K* and *r* are regression coefficients. These analyses were conducted for mean annual productivity per grid cell during 2000–2015, and the mean productivity per grid cell of the highest 2, 4 and 8 years and the lowest 2, 4 and 8 years out of the 16 years separately.

We conducted multiple regression analyses using different fitting sequences to compare the relative effects of different variables on primary productivity: (1) leaf size *vs*. AET; (2) leaf size *vs*. LAI; (3) AET, leaf size and LAI and (4) AET, leaf size and soil characteristics. The independent and joint effects of different variables were compared using analysis of variance (ANOVA). We also conducted multiple regression analyses and ANOVA to explore the effects of clade ages on leaf size–primary productivity relationships.

To assess whether climate affects primary productivity directly or indirectly via leaf size and LAI, we built three structural equation models (SEMs) hypothesising that (1) climate affects leaf size and they both affect productivity; (2) leaf size affects LAI and they both affect productivity; and (3) climate affects both leaf size and LAI, and all the variables affect productivity (Figs. S4.6, S4.9, S4.11). SEMs were built with the R package ‘*piecewiseSEM*’. Missing paths in model 3) were detected using the ‘*sem.missing.paths*’ function. As these SEMs were saturated, we did not use goodness‐of‐fit statistic for model evaluation but directly tested the significance of path coefficients.

### Transfer functions to predict ecosystem primary productivity

To explore the use of leaf size for predicting/reconstructing primary productivity in other regions/times, we built linear and nonlinear transfer functions based on the strong empirical relationships of leaf size with MODIS‐derived GPP and NPP in China (Table 1, Table S6.1) and North America (Table S6.2). To evaluate the generality of these transfer functions, we applied those established in China to leaf size in North America and vice versa. Scatter plots between the predicted and MODIS‐derived GPP and NPP were generated and compared with the 1:1 lines.

**Table 1:** Transfer functions between primary productivity and leaf size based on data from China. The transfer functions were built with data from China using the model Y = 1/r*(*a*‐ln(*K*/X‐1)); *R* ^2^‐values of the best models are in bold. All relationships are significant at *P* < 0.001

*y*	*x*	K	*α*	*r*	*R* ^2^	No. of cells	SE	SE%
GPP	Length	11.602	1.470	0.002	0.83	3333	220.994	8.139
	Width	5.228	1.558	0.002	0.78	3332	251.688	9.270
	Length‐width product	50.641	2.985	0.003	0.78	3332	248.658	9.162
NPP	Length	11.500	1.567	0.004	0.79	3333	119.643	8.062
	Width	5.208	1.669	0.005	0.74	3332	132.578	8.934
	Length‐width product	50.567	3.089	0.006	0.76	3332	127.389	8.588

The transfer functions are $y\;=\;\frac{1}{r}\left(\alpha -\ln\left(\frac{K}{x}-1\right)\right)$, where *x* < K and K, *α* and *r* are the three parameters of the model. x, leaf size measures (length, width or length‐width product); y, ecosystem primary productivity (GPP or NPP); *R^2^*, coefficient of determination of model; # of cells, numbers of grid cells included in the calculation of nonlinear models (with x < K); SE, standard error of estimate ($\text{SE}\;=\;\sqrt{\frac{\sum {\left(y_{\text{est}}-y_{\text{real}}\right)}^{2}}{n-1}}$); SE%, standard error ratio of estimate (${\text{SE}}_{R}\;=\;\frac{\text{SE}}{y_{real(\max)}-y_{real(\min)}}\times 100\%$) (Traiser *et al.* 2005). See Table S6.1 for parameters of other models.

All statistical analyses were conducted in R v3.3.5 (http://www.r‐project.org/).

## RESULTS

### Geographical patterns of leaf size and their relationships with climate

**Figure 1:**
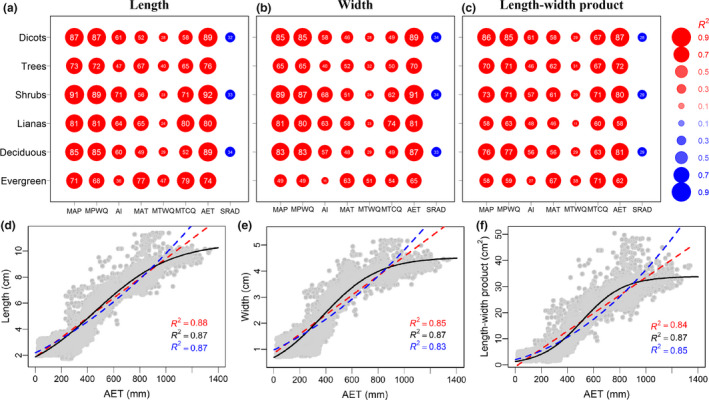
The explanatory power of environmental variables on the geographical patterns of leaf size (a–c) and the relationship between leaf size and annual evapotranspiration (d–f) in China. From left to right, the three columns represent leaf length, leaf width and leaf length‐width product (with a correction factor of 2/3). In (a), (b) and (c) each dot represents a relationship between an environmental factor and a leaf size measure evaluated with logistic regression. The sizes of the dots together with the numbers on them represent *R* ^2^ (expressed as percentages, %); red and blue colours represent positive and negative correlations respectively. The *P* values of all the regressions were evaluated with a modified *t*‐test (Dutilleul *et al.* 1993), and only dots with *P* values < 0.001 were included because of the multiple pairwise tests of leaf size–climate correlations (α = 0.05/48). Corresponding full scatter plots are presented in Figures S1.6–S1.8 and for the corresponding *R* ^2^ and *P* values of those relationships are presented in Table S1.5. Note: in (a), (b) and (c) the relationships between leaf length, leaf width and leaf length‐width product, respectively, with from left to right mean annual precipitation (MAP, mm), precipitation of wettest quarter (MPWQ, mm), aridity index (AI, mm), mean annual temperature (MAT, °C), mean temperature of warmest quarter (MTWQ, °C), mean temperature of coldest quarter (MTCQ, °C), annual actual evapotranspiration (AET, mm) and annual solar radiation (SRAD, kJ cm^−2^ day^−1^) are given from top down for all woody dicots, trees, shrubs, woody lianas, deciduous dicots and evergreen dicots). In (d), (e) and (f) logistic (black solid lines) and linear (red‐dashed lines) regressions are shown together with corresponding *R*‐values. The vertical axis represents average leaf length (d), leaf width (e) and length‐width product (f) of all woody dicots, and the horizontal axis represents AET.

As expected, community mean leaf size decreased from southern China to the north and northwest (Fig. S1.1). Mean leaf size in China increased with temperature, precipitation and AET, but decreased with SRAD (Figs. 1, S1.6–S1.8). Moreover, the relationships between leaf size and climate were highly consistent across life forms (Fig. 1; Appendix S1).

Among the tested variables, AET had the highest overall and independent effects on leaf size variations for all species and most life forms in China and North America (Fig. 1, Figs S1.6‐S1.9, S6.2). Specifically, logistic regressions indicated that AET overall explained 87–89% of the spatial variation in leaf size for all Chinese woody species (Fig. 1; Table S1.5). HPAs showed that the independent effects of precipitation were lower than those of AET (Fig. S1.9). Temperature had much lower overall and independent effects on leaf size variation than precipitation (Fig. S2.2).

### Relationships between leaf size and ecosystem primary productivity

**Figure 2:**
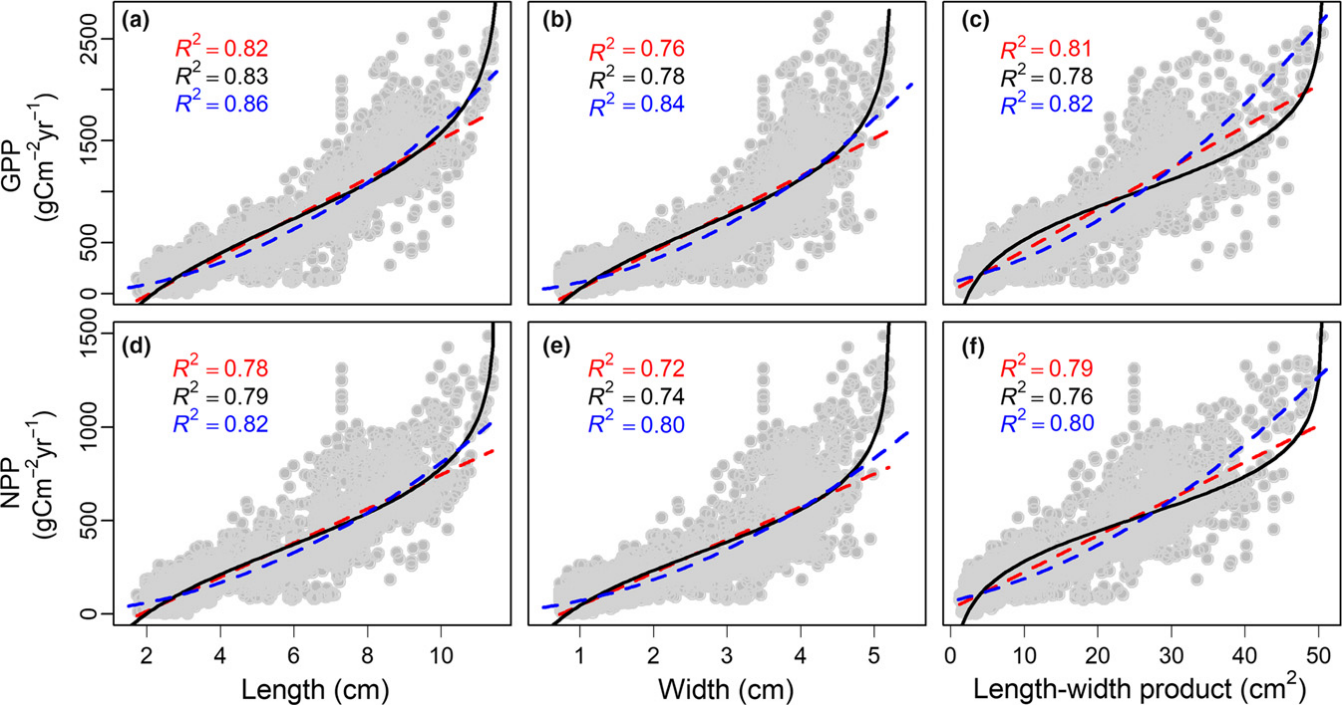
Variation in primary productivity in relation to leaf size in China. From left to right, the three columns represent average leaf length (cm), leaf width (cm) and leaf length‐width product (cm^2^) of woody dicots. The first and second rows represent gross primary productivity (GPP, gC m^−2^year^−1^) and net primary productivity (NPP, gC m^−2^year^−1^) respectively. GPP and NPP were estimated from a widely used MODIS product (See Methods for more detail). The regression lines and *R* ^2^ values were estimated with both logistic (black lines and numbers for the logistic function y = 1/*r *× (*a*‐ln(*K*/*x*‐1)) and two linear regressions (blue lines and numbers of the function sqrt(y) = *a* + *b *×* x* and red dashed lines and numbers for the function y = *a* + *b *× *x*). Note that only when leaf size values are less than the estimated K can logistic regressions be used to predict GPP and NPP

Community mean leaf size was strongly correlated with MODIS‐derived GPP and NPP for both China and North America (Fig. 2, Fig. S6.3). Specifically, leaf size explained 76–86% of the spatial variation in GPP and 72–82% in NPP in China (Fig. 2). These strong relationships were verified with flux‐based GPP and CASA‐simulated NPP (Fig. S4.2). Leaf size was also significantly correlated with biomass‐estimated NPP in Chinese forests (Fig. S4.3). However, these relationships were weaker than those between leaf size and MODIS‐derived NPP. Biomass‐estimated NPP and MODIS‐derived NPP were, however, even more weakly correlated with one another (Table S4.1), which indicates a challenge of reconciling ground sampling done at fine scales (and with varying site selection criteria) with satellite‐derived or modelled productivity at spatial domains orders of magnitudes larger. We further found that explanatory powers of mean leaf size on primary productivity decreased from years with high GPP or NPP (‘good’ water‐energy conditions) to years with low GPP or NPP (Fig. S4.4; Table S4.2), suggesting that inter‐annual variation in primary productivity might bring uncertainties in the relationships of leaf size with primary productivity. However, these uncertainties tend to be low because even in years with low GPP or NPP, leaf size still explained 68–81% of the spatial variations in GPP and NPP.

**Figure 3:**
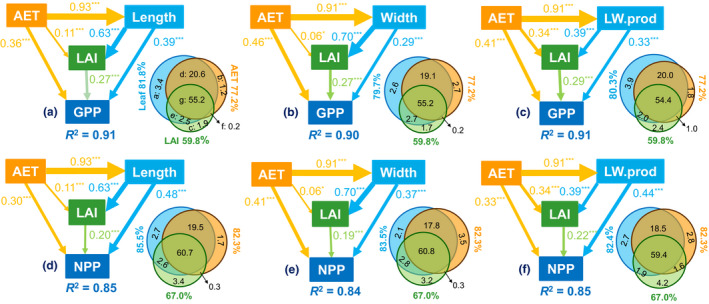
The linkages among climate, leaf size, leaf area index (LAI) and ecosystem primary productivity in China. (a)–(f): Standardised results of the final structural equation models (SEMs) to examine the potential pathways of links among variables; along with Venn diagrams (VDs) to show the relative contributions of climate, leaf size and LAI to variation in primary productivity. Note: AET, annual actual evapotranspiration; Length, community mean leaf length; Width, community mean leaf width; LW.prod, community mean leaf length‐width product; GPP, MODIS‐derived gross primary productivity; NPP, MODIS‐derived net primary productivity. Both GPP and NPP were square root transformed. The numbers in each pathway of SEMs show the standardised coefficients with significance (***P < 0.001; **P < 0.01; *P < 0.05). The numbers in VDs show the explained percentage of total variation (%): the independent effect of leaf size (a), AET (b) and LAI (c), and the joint effect of leaf size and AET (d), leaf size and LAI (e), AET and LAI (f) and all the three variables (g). The numbers around the circles are total effect of leaf size (in blue), AET (in yellow) and LAI (in green). The sizes of the circles are scaled to the *R* ^2^ values of the corresponding regression models.

Multiple regression analyses showed that the independent effects of leaf size on MODIS‐derived GPP and NPP were larger than (or in some cases comparable with) those of AET, LAI and soil characteristics (Fig. 3, Figs S4.5, S4.7, S4.10). Furthermore, the SEMs indicated that direct effects of AET on GPP and NPP were generally weaker than indirect effects via leaf size and LAI (Fig. 3, Fig. S4.11; Table S4.5).

### Influence of evolutionary age on the leaf size–environment relationships

For Chinese data, we did not find significant phylogenetic signals in mean leaf size per family or genus for all dicots and most life forms (Table S3.1), suggesting that leaf size is not strongly phylogenetically conserved but evolutionarily labile at both family and genus levels, and leaf size variation reflects adaptations to contemporary climate. Nevertheless, small but significant negative interactions between mean clade age and leaf size upon primary productivity suggest that older assemblages showed weaker relationships between leaf size and productivity (Tables S3.2–S3.4), indicating some evolutionary constraints on leaf size adaptation to environment.

### Using leaf size to predict ecosystem primary productivity

**Figure 4:**
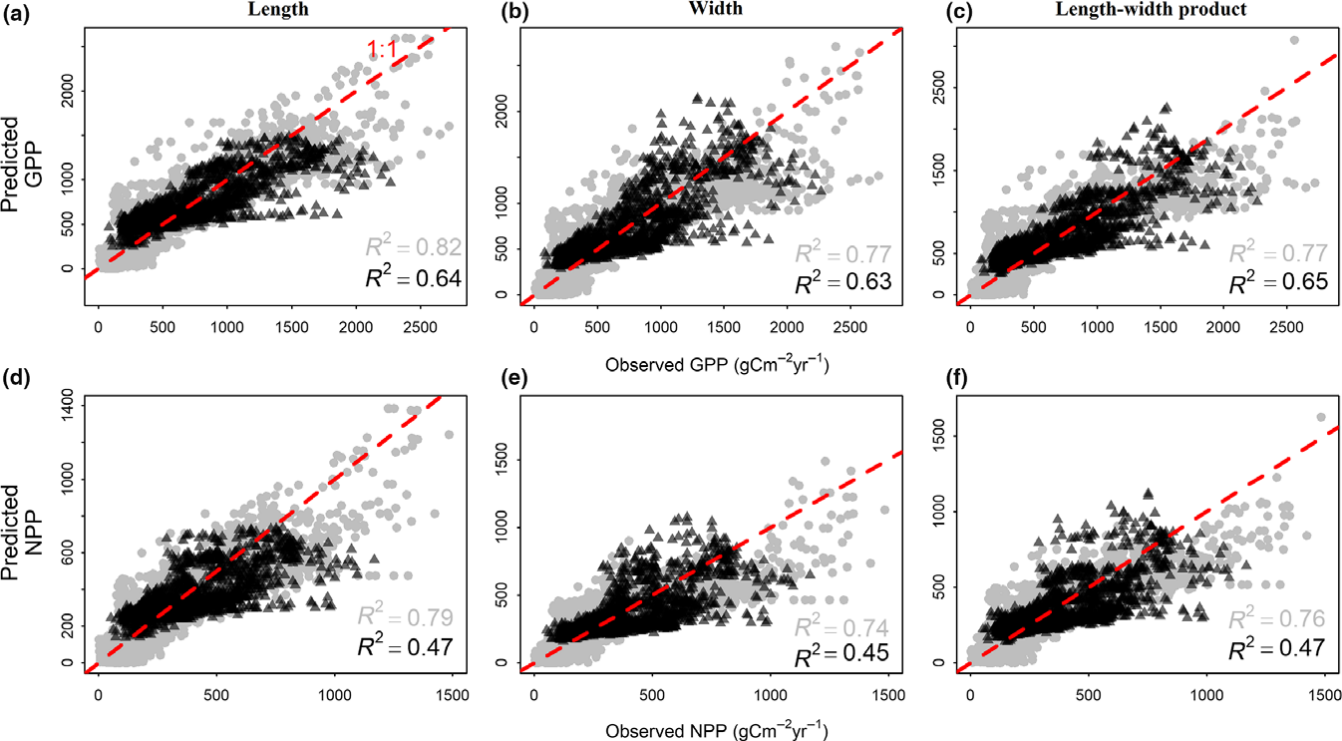
Comparison between the MODIS‐derived ecosystem primary productivity and the predictions by the transfer functions of China using leaf size in China and in North America. From left to right, the three columns represent leaf length (cm), leaf width (cm) and leaf length‐width product (cm^2^). The first and second rows represent gross primary productivity (GPP, gC m^−2^year^−1^) and net primary productivity (NPP, gC m^−2^year^−1^) respectively. Black triangles and numbers represent North America while grey dots and numbers represent China as the background. The red dashed lines represent the 1:1 reference relationship. See Table 1 for parameters of the transfer functions. Leaf size in North America was estimated with BIEN range maps; similar results based on other distribution data are presented in Supplementary Information Appendix S6 (Figures S6.4–S6.5)

**Figure 5:**
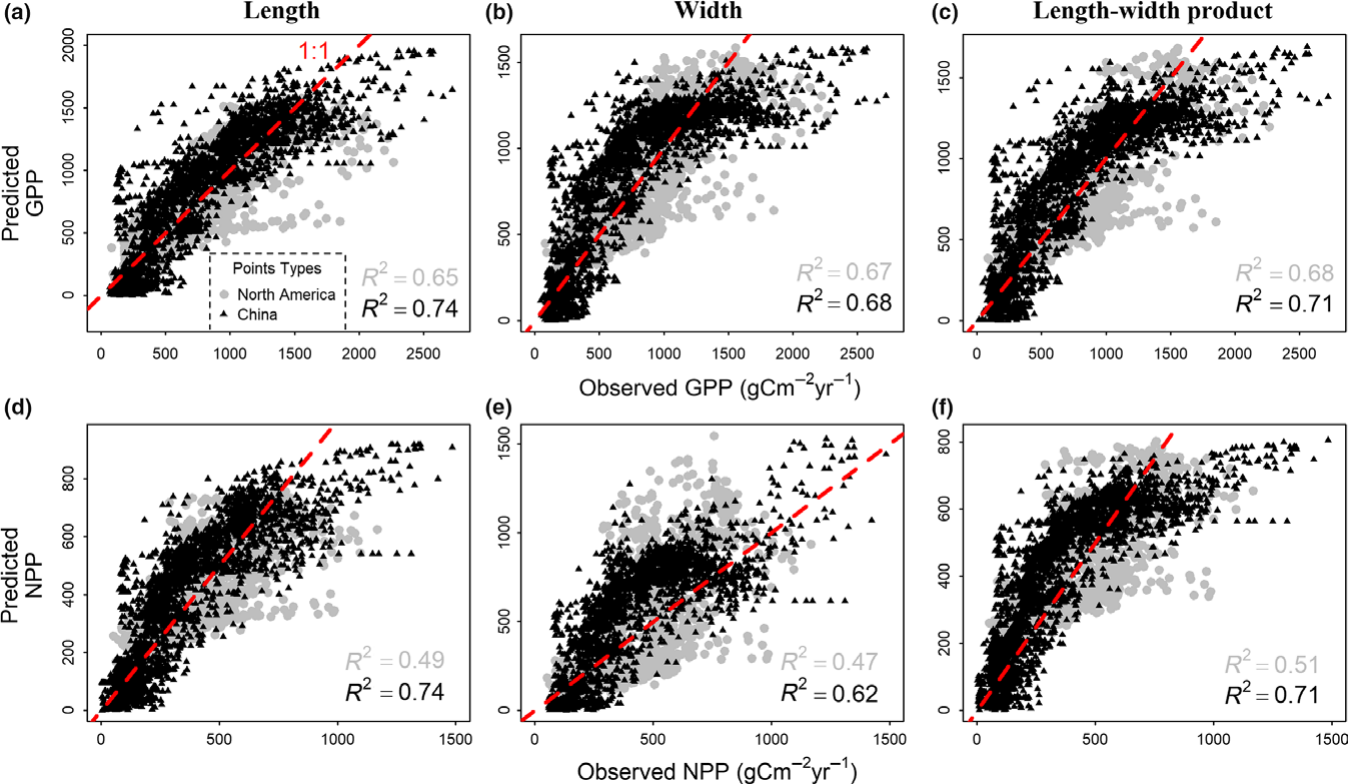
Comparison between the MODIS‐derived ecosystem primary productivity and that predicted by the transfer functions of North America using leaf size in North America and China. Black triangles and numbers represent China while grey dots and numbers represent North America as the background (compare with Figure 4). The meaning of axes, lines and legends is the same as in Figure 4. See Table S6.2a for parameter values. Leaf size in North America was estimated with BIEN range maps; similar results based on other distribution data are presented in Supplementary Information Appendix S6 (Figure S6.6)

GPP and NPP predicted by the transfer functions in China were strongly correlated with MODIS‐derived GPP and NPP in North America (Fig. 4, Figs S6.4–S6.5). Similarly, the transfer functions based on North American data also predicted well the primary productivity in China (Fig. 5, Fig. S6.6). Notably, the performance of transfer functions built from the North American dataset were weaker compared to those built from the Chinese dataset (i.e. relatively lower model *R^2^* and slight underestimation in regions with high productivity, Fig. 5), which might be partly due to the difference in spatial scales and lower data density in North America. Nevertheless, the consistent results between China and North America suggest that leaf size can be used as an indicator of primary productivity, at least in the northern temperate and subtropical woody ecosystems.

## DISCUSSION

Based on newly compiled morphological and distribution data of woody dicots in China and North America, we quantified the geographical variation in community mean leaf size across a large spatial extent and show how it is related to climate and primary productivity. We found that among the tested variables, leaf size was most strongly correlated with AET, NPP and GPP, and these relationships did not differ among life forms. Furthermore, the transfer functions based on the leaf size–primary productivity relationships in China provided reasonable predictions of primary productivity in North America and vice versa. These results advance our understanding of trait–ecosystem function biogeography and also suggest that community mean leaf size could be used to statistically predict modern or to reconstruct terrestrial palaeo‐primary productivity from living plant samples or leaf fossils respectively. As noted below, such predictions may be influenced both by direct influence of leaf size on biological processes and by co‐variation of leaf size with other climate and ecosystem properties that might influence productivity in parallel.

### Influences of climate on leaf size

Understanding climatic controls on leaf size helps to explore trait variations and their consequences for ecosystem properties at large scales. The key driver of leaf size variation is AET, which represents both precipitation and energy to convert that moisture to vapour. This is consistent with a global study that found leaf size was smaller if either precipitation or energy driving AET were low (Wright *et al.* 2017).

Previous studies on other plant traits, for example leaf margin (Li *et al.* 2016), flowering time (Du *et al.* 2015) and seed mass (Moles *et al.* 2005), found that these traits are constrained by phylogeny and their variations are influenced by plant life forms. Phylogenetic conservatism of traits may limit responses of plants to climate variability or change (Davies *et al.* 2013). For example previous studies found that leaf‐margin traits of woody dicots have significant phylogenetic signals, which constrain the relationships between leaf‐margin traits and temperature (Little *et al.* 2010; Li *et al.* 2016). However, our study did not find significant phylogenetic signals in leaf size at both the family and genus level, suggesting that geographical variation in leaf size is mainly determined by climate filtering (Wright *et al.* 2017) and short‐time adaptive evolution rather than deep phylogenetic history (Ackerly 2009; Milla & Reich 2011). Indeed, although we did find slightly stronger leaf size–environment relationships in areas with species belonging to younger (i.e. recently evolved) clades than in areas with species belonging to older (i.e. earlier evolved) ones, the phylogenetic constraints on leaf size adaptation to newer climates were very weak (Table S3.2–S3.4). The lack of phylogenetic signals in leaf size thus may also be one of the reasons for its strong relationships with contemporary climate. In summary, our findings suggest that leaf size can respond to climate change quickly.

### Correlation between leaf size and ecosystem primary productivity

It is important to recognise that community mean leaf size and climate may both play important roles in determining ecosystem primary productivity, and that the strong relation between the two limits our ability to know the relative impact of each independently. The strong correlation between leaf size and primary productivity may at least in part be due to a positive contribution of large leaf size on both leaf photosynthesis and LAI, and thus on canopy photosynthesis (Reich 2012; Michaletz *et al.* 2016; Wright *et al.* 2017). Leaf size may influence canopy photosynthesis in at least two ways. First, leaf size could influence photosynthetic rate through its influence on leaf surface temperature. Previous studies suggest that plants maintain leaf temperature for particular climatic conditions that maximise carbon uptake from photosynthesis and reduce water loss via transpiration (Parkhurst & Loucks 1972; Yates *et al.* 2010; Michaletz *et al.* 2016). Several studies show that leaf thermoregulation is related to leaf size (Yates *et al.* 2010; Leigh *et al.* 2017; Fauset *et al.* 2018). Particularly, leaf surface temperature is strongly influenced by the thickness of leaf boundary layer that is positively correlated with leaf size (Schuepp 1993; Michaletz *et al.* 2016; Leigh *et al.* 2017). Second, leaf size is linked to effective photosynthetic area via total leaf area of plants, and thus contributes to higher canopy photosynthetic capacity and biomass accumulation (Enquist *et al.* 2007; Reich 2012). However, leaf size affects primary productivity not only by influencing effective photosynthetic area but also by influencing photosynthetic rate. This may explain why we found that leaf size was a better predictor of primary productivity than LAI, although co‐variation of large leaf size with optimal conditions for productivity might also be involved. Further experimental and modelling studies are needed to quantify the influence of leaf size on both photosynthetic rate and area, and the mechanisms behind the functional relationships between leaf size and primary productivity.

The strong correlation between leaf size and primary productivity may be also partly due to the similarity in how climate influences leaf size of individuals and total leaf area of canopies. Mechanistically, leaf size varies with the ways that climate influences leaf energy budgets and how natural selection acts to maximise leaf carbon budgets (Parkhurst & Loucks 1972; Wright *et al.* 2017). Warmer, wetter and less seasonal environments also influence plant hydraulics (Pfautsch *et al.* 2016) and permit increased canopy size (reflected by higher LAI), and hence, increased annual primary productivity (Gower *et al.* 1999; Reich 2012). These findings suggest that covariation in leaf size and LAI can increase the statistical coupling of leaf size with primary productivity. Moreover, a recent experimental study found that at the small scale of plots (20 × 20 m) leaf area drove more ecosystem functions (e.g. those related to aboveground stocks) than other most frequently assessed traits (van der Plas *et al.* 2019). The degree to which leaf size alone can mechanistically drive productivity, however, remains unclear. Unravelling this would be critical to any effort to incorporate leaf size into process‐based land surface models, but is less important for using palaeo‐leaf size as a proxy for palaeo‐primary productivity reconstruction.

In our analyses, community mean leaf size was calculated from the presence/absence data. Recent meta‐analyses indicate that community mean traits weighted by abundance normally strengthen trait–climate relationships in most cases (Newbold *et al.* 2012; Griffin‐Nolan *et al.* 2018), but in some cases abundance‐weighted mean traits had similar, or even weaker trait–climate relationships compared with unweighted ones (Pakeman *et al.* 2008; Cornwell & Ackerly 2009). Studies on trait–abundance relationships indicated that species with resource‐acquisitive traits (i.e. large leaves) tended to have high abundance in warm and humid regions (Adler *et al.* 2013), while species with water‐conservative traits (i.e. small and thick leaves) tended to have high abundance in arid regions (Cornwell & Ackerly 2010). We speculated that large‐leaved species might have higher abundance in regions with higher productivity that also harbour more large‐leaved species, and hence an abundance‐weighted approach might improve leaf size–primary productivity relationships. Moreover, our analyses did not include intraspecific variation in leaf size, which might also introduce uncertainties in leaf size–primary productivity relationships. Data on species abundances and intraspecific trait variations at large spatial scales are needed to account for these uncertainties in future macroecology studies.

Finally, we point out that at the scale of local communities, diversity in leaf size likely contributes additional explanatory power to primary productivity, as positive effects of such diversity on productivity have been found in multiple biodiversity experiments in grasslands (Roscher *et al.* 2012) and forests (Huang *et al.* 2018). However, such diversity effects may be less likely at the macro‐ecological scale in our study.

### Using leaf size to reconstruct palaeo‐primary productivity

Palaeo‐primary productivity is a key index for understanding ecosystem dynamics in response to long‐term historical climate change. To reconstruct terrestrial palaeo‐primary productivity, appropriate surrogates are needed. Given that leaf size of woody species with different life forms has consistently strong relationships with primary productivity, the transfer functions based on current relationships might not be influenced by species composition in different floristic regions. We therefore suggest that the relatively high availability of leaf fossils and leaf samplings of regional floras or communities provide a promising practical surrogate to reconstruct woody ecosystem primary productivity for past environments (Table S6.4–S6.5). Exploring preservation biases of leaf fossils would be helpful to improve the precision of this method.

Our study demonstrates the generality of leaf size–primary productivity relationships in the northern temperate and subtropical woody ecosystems. Further studies are needed to test the generality and application of this method across wider biogeographical regions (e.g. extending to the Southern Hemisphere) and at multiple spatio‐temporal scales, and to decipher the portion of these relationships that are due mechanistically to leaf size per se.

## AUTHOR CONTRIBUTIONS

Z.W. and Y.L. designed the research; Y.L., Z.W., B.M., T.L., X.X., X.S. and Z.T. collected the data; Y.L. performed the analyses with substantial contributions from Z.W., B.S., P.R., N.S. and B.E.; all authors discussed the results; Y.L., Z.W., P.R., B.S., X.F. and B.E. led the writing with contributions from all co‐authors.

## Supporting information

Supplementary Material

## Data Availability

The data used in this study are available in the Dryad Digital Repository: https://doi.org/10.5061/dryad.kwh70rz0q.
