# Supplementary material for: Leaf size of woody dicots predicts ecosystem primary productivity
Source: Ecol Lett. 2020 Apr 6;23(6):1003–13. doi: 10.1111/ele.13503 (PMC7384143; doi:10.1111/ele.13503)
Supplement: Supplementary file 1 — Supplementary Material [file ELE-23-1003-s001.pdf]

Supplementary Information for

Leaf size of woody dicots predicts ecosystem primary productivity

Yaoqi Li[1], Peter B. Reich[2,3], Bernhard Schmid[4], Nawal Shrestha[1,5], Xiao Feng[6], Tong Lyu[1], Brian S. Maitner[7], Xiaoting Xu[8,1], Yichao Li[1], Dongting Zou[1], Zheng-Hong Tan[9], Xiangyan Su[1], Zhiyao Tang[1], Qinghua Guo[10], Xiaojuan Feng[10], Brian J. Enquist[6,11], Zhiheng Wang[1*]

[1] Institute of Ecology and Key Laboratory for Earth Surface Processes of the Ministry of Education, College of Urban and Environmental Sciences, Peking University, Beijing 100871, China

[2] Department of Forest Resources, University of Minnesota, St. Paul, MN 55108, US

[3] Hawkesbury Institute for the Environment, Western Sydney University, Penrith, NSW 2751, Australia

[4] Department of Geography, Remote Sensing Laboratories, University of Zurich, Winterthurerstrasse 190, 8057 Zurich, Switzerland

[5] Institute of Innovation Ecology, Lanzhou University, Lanzhou 730000, China

[6] Institute of the Environment, University of Arizona, Tucson, Arizona 85721, USA

[7] Department of Ecology and Evolutionary Biology, University of Arizona, Tucson, AZ 85721, USA

[8] Key Laboratory of Bio-Resource and Eco-Environment of Ministry of Education, College of Life Sciences, Sichuan University, Chengdu 610065, Sichuan, China

[9] College of Environment and Ecology, Hainan University, Haikou, Hainan 570228, China

[10] State Key Laboratory of Vegetation and Environmental Change, Institute of Botany, Chinese Academy of Sciences, Beijing 100093, China

[11] The Santa Fe Institute, Santa Fe, NM 87501, USA

Correspondence should be addressed to: Zhiheng Wang

Email: zhiheng.wang@pku.edu.cn

This file includes:

Data supplement

Appendix 1 to Appendix 6

Figures S1.1 to S6.6

Tables S1.1 to S6.5

References for SI reference citations

Data supplement

Leaf size, life forms and distributions of woody dicots in China. In this study, we focused on woody dicots (including Eudicots and Magnoliids) and did not include gymnosperms and woody monocots (mostly bamboos) following previous studies (Traiser *et al.* 2005; Peppe *et al.* 2011). Gymnosperms mainly have acicular or phyllade leaves, which is different from those of dicotyledons and hence may bring uncertainties into the estimation of mean leaf widths and leaf length-width products of regional floras. We compiled the leaf length and width ranges (i.e. minimum and maximum values) of mature individuals for each species from the *Flora of China* (English version, *http://www.efloras.org/flora_page.aspx?flora_id=2*; Chinese version, *http://www.iplant.cn/frps*; accessed January 2014) and the *Chinese Virtual Herbarium* (*http://www.cvh.ac.cn/frps/* , accessed January 2014). Finally, our database contained 10,480 woody dicots in total, with 9,855 species (ca. 94%) having leaf length data and 9,695 species (ca. 93%) having leaf width data. In general, leaf sizes of a species recorded in floras reflect variation of mature leaves across its populations and are estimated from multiple specimens and sometimes field observations. During specimen collection, sampling branches with mature leaves, flowers and fruits in the upper canopy are optimal, but the challenge of sampling high in a canopy means many leaves were likely sampled in lower, more shaded positions than preferred. Considering the large number of species and the low contribution of tall species to local floras (Fig. S1.3), our following estimation of mean leaf size should not be substantially affected by potentially and unknown biased sampling.

Previous studies found that leaf area of a species is positively linearly correlated with its leaf length x leaf width product across various environments (Cristofori *et al.* 2007; Rouphael *et al.* 2010). A correction factor of 2/3 or 3/4 is often used to account for leaf area of the generally elliptical shape of leaves (Cain & Castro 1959; Wilf *et al.* 1998). As the correction factor is a

constant (i.e., 2/3), it does not affect the evaluation of the correlation between leaf area and climate nor does it influence the performance of leaf size–productivity transfer functions. We therefore included the results based on length x width product with a correction of 2/3 as a surrogate of leaf area in the main text. Note that this is not intended as an estimate of true average leaf area, but instead, simply as a quantitative scaling metric. In total, we could calculate leaf length x leaf width product for 9,677 woody dicots (ca. 92%) in our database. In the following analyses, all the three measures of leaf size were used: leaf length, leaf width and length-width product (with a correction of 2/3).

We also collected information on the life forms (trees, shrubs and lianas; deciduous and evergreen) of the studied species from the *Flora of China*. Species with multiple or uncertain life forms were not included in the corresponding analyses. Finally, our database contained 2,939 trees, 4,934 shrubs and 913 woody lianas, and 3,477 deciduous and 4,485 evergreen species.

Species distribution maps of all these species were extracted from the *Atlas of Woody Plants in China: Distribution and Climate* (Fang *et al.* 2011). The species distribution data in this atlas were compiled from all published national- and provincial-level floras, as well as a large number of local floras and inventory reports across the country (Wang *et al.* 2011). The obtained county-level distribution maps were then transformed into an equal-area grid of 50 × 50 km to eliminate the influence of area on the estimation of species diversity (see Wang *et al.* 2011 for more details).

Leaf size and life forms of woody dicots in North America. The data of leaf length, leaf width, and life form for all species in North America north of Mexico (i.e. Canada and continental United States) were compiled from the *Flora of North America* (*FNA*, *http://efloras.org/flora_page.aspx?flora_id=1*, accessed June 2017), supplemented with data

from the *Botanical Information and Ecology Network* (*BIEN*) (Goldsmith *et al.* 2016; Maitner *et al.* 2018). Species names from different data sources were corrected according to the *Taxonomic Name Resolution Service* (Boyle *et al.* 2013). We then extracted leaf size data for only woody dicots and removed all introduced species. Ultimately, 2,374 woody dicots were included, in which 2,037 species had leaf size data. Since only parts of *FNA* are published online, the data for North America were not as complete as for China.

Species distributions were compiled from two different sources: a) occurrences and range maps were collected from *BIEN* (accessed January 2018; see Appendix 5) and then rasterized into 100 × 100 km grids (considering the resolution of available data); b) state- or province-level distributions were compiled from *FNA* and *USDA* plant database (*https://plants.usda.gov/java/*, accessed June 2017). All data from BIEN were downloaded via the R package "BIEN" (Maitner *et al.* 2018) and those from *FNA* and *USDA* were downloaded directly.

Environmental data. Mean annual temperature (MAT), mean annual precipitation (MAP), mean temperature of warmest quarter (MTWQ), mean temperature of coldest quarter (MTCQ) and precipitation of wettest quarter (MPWQ) of the year as well as monthly mean temperature and precipitation were obtained from the *WorldClim* database (*http://www.worldclim.org/*, Version 1.4, accessed January 2014) at a spatial resolution of 1 × 1 km (Hijmans *et al.* 2005). The monthly mean solar radiation between 1970 and 2000 were obtained from the *WorldClim* database (Version 2.0) at a spatial resolution of 10 minutes. We resampled each environmental layer within China into 50 × 50 km resolution using "zonal" Statistics tool in ArcGIS 10.0. Similarly, we resampled environmental layers in North America.

Monthly mean temperature and precipitation were used to calculate annual actual evapotranspiration (AET) and annual potential evapotranspiration (PET) following the method of

Thornthwaite & Hare (1955). AET reflects the amount of water that plants can actually use, and is strongly correlated with the ecosystem primary productivity (Garbulsky *et al.* 2010; Yuan *et al.* 2010). Therefore, AET has been used as a surrogate of ecosystem productivity (Lieth 1975).

In our preliminary analyses, we used water deficit (WD = PET–AET) and two different definitions of AI, AI = MAP/PET (United Nations Environment 1992) and AI = MAP–PET (Klein *et al.* 2015), to represent the aridity of a region. The results showed that AI = MAP–PET was the best predictor of leaf size among these three variables (Fig. S1.2). Similarly, several studies found that AI = MAP–PET could significantly influence tree distribution (Piedallu *et al.* 2013), canopy height and leaf expansion (Klein *et al.* 2015), and thus could be the best proxy representing water availability for trees. Therefore, only AI = MAP–PET was used in the following analysis.

Furthermore, we collected soil data from *SoilGrids* (*https://files.isric.org/soilgrids/data/*, accessed by January 2019). The soil dataset was at 250 m resolution on the global scale (Hengl *et al.* 2017), compiled using machine learning methods based on soil survey plots from *Harmonized World Soil Database*, MODIS-derived monthly ground surface temperature and monthly precipitation data from *WorldClim*. We used three variables to describe soil characteristics, namely soil pH, soil organic carbon content (Soil OCC, g/kg) and soil cation exchange capacity (Soil CEC, c mol+/kg) in the top 30-cm soil layer, which have been used to represent soil characteristics in previous studies (Maire *et al*. 2015; Bruelheide *et al*. 2018). We could not obtain data of soil nitrogen and phosphorus content that fitted our study requirements. We conducted principal component analysis (PCA) with the three soil variables and extracted the first principal component, which explained > 70% of the total variance, to represent edaphic variation.

For comparison with MODIS-derived GPP and NPP, we also obtained a flux-based GPP dataset from 1982–2015 at 0.1° resolution estimated with the Model Tree Ensemble (MTE) algorithm using data from 40 flux sites over China (Yao *et al.* 2018) and an improved NPP dataset from 1982–2015 at 8-km resolution estimated with the Carnegie-Ames-Stanford Approach (CASA) using data from 2480 Chinese climate stations and normalized difference vegetation index data (CASA-simulated NPP) (Feng *et al.* 2019). We also obtained biomass-estimated NPP data for 1099 forest stands in China from a recent publication (Michaletz *et al.* 2014), in which production of different plant parts was estimated from biomass–productivity allometric relationships and then summed up.

To compare the effects of leaf size and LAI on primary productivity, we obtained LAI data from *GLOBMAP LAI* (*http://www.modis.cn/globalLAI/GLOBMAPLAI_Version2/*, accessed June 2017) with time resolutions of half months (1981–2000) or 8 days (2000–2015). To generate this LAI product, MODIS and Advanced Very High Resolution Radiometer data were fused with a pixel-by-pixel approach to generate a consistent long temporal range (Liu *et al.* 2012). The mean LAI in July for the 35-year period was estimated at the spatial resolution of 50 × 50 km in ArcGIS 10.0.

Supplementary Information for Li *et al.* (2020)

***Appendix* 1 The relationships of spatial variations in leaf size with climate and the influence of life form.**

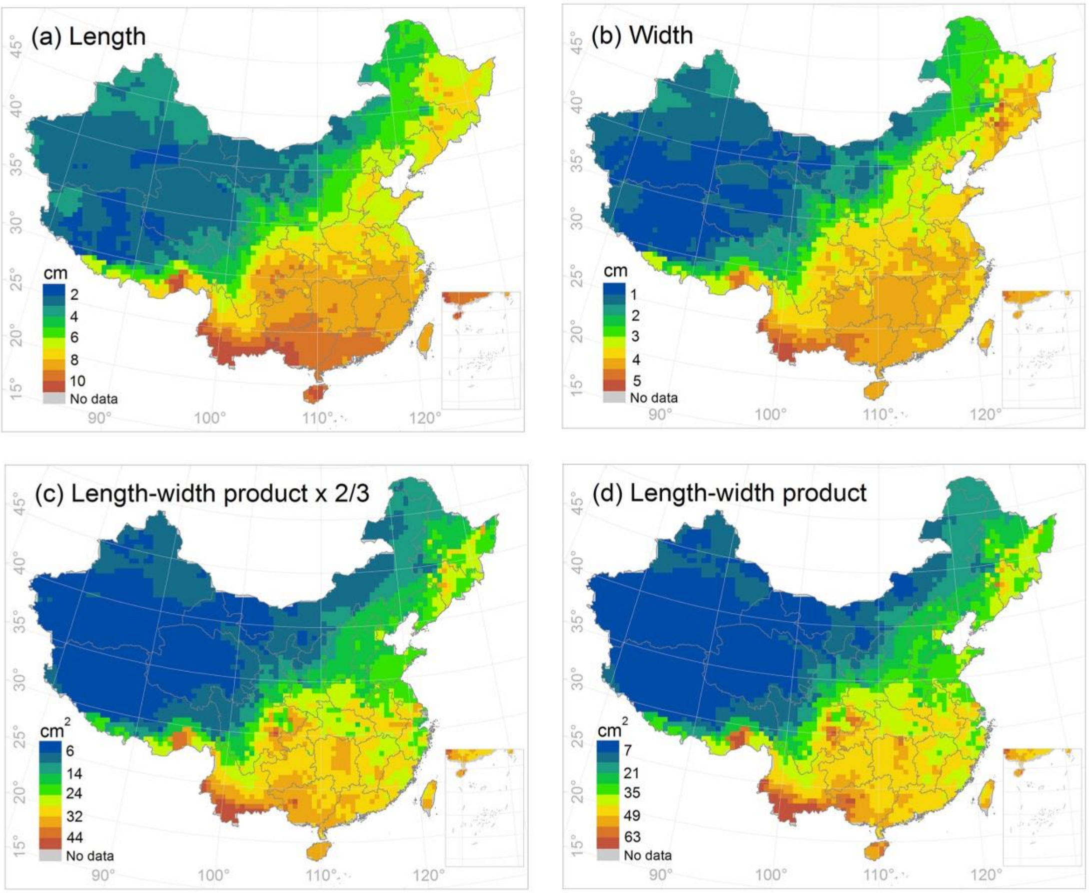

Figure S1.1 The geographical patterns in average leaf size of all woody dicot species in China. a), leaf length (cm); b), leaf width (cm); c), the product of leaf length and width (cm^2, with a correction factor of 2/3); d), the product of leaf length and width (cm^2, without a correction factor). Totally 3,794 grid cells with a spatial resolution of 50 × 50 km are shown. Values increase from blue to red.

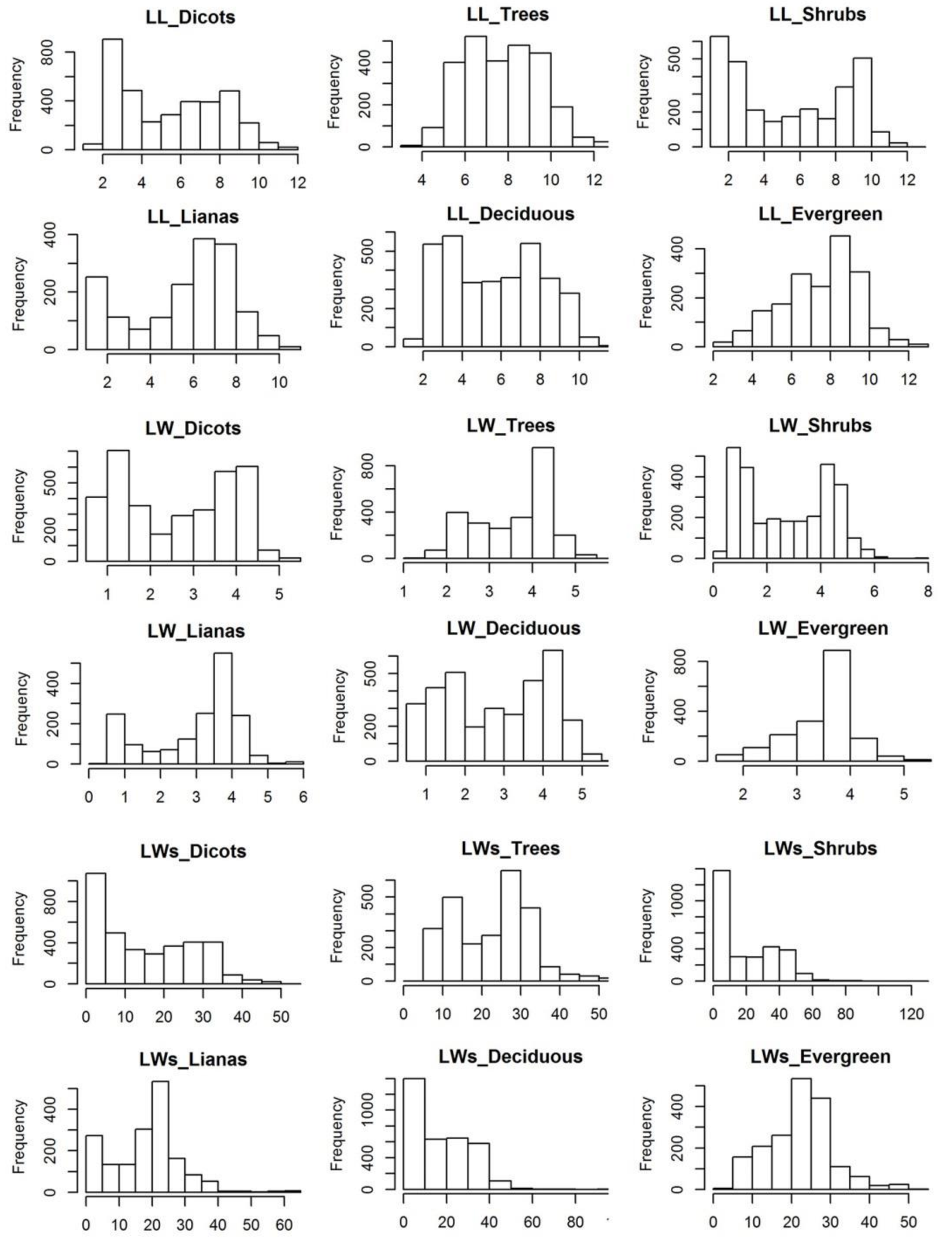

Figure S1.2 Histograms of leaf size measures. The frequency distributions of mean leaf size measures per grid cell are shown. The first two rows represent the average leaf length (LL, cm) for species with different life forms (from left to right: all dicots, trees, shrubs, woody lianas, deciduous dicots and evergreen dicots). The second and third pairs of two rows, respectively, represent the average leaf width (LW, cm) and length-width product (LWs, cm^2; with a corroction factor of 2/3; and hereafter) for each life form.

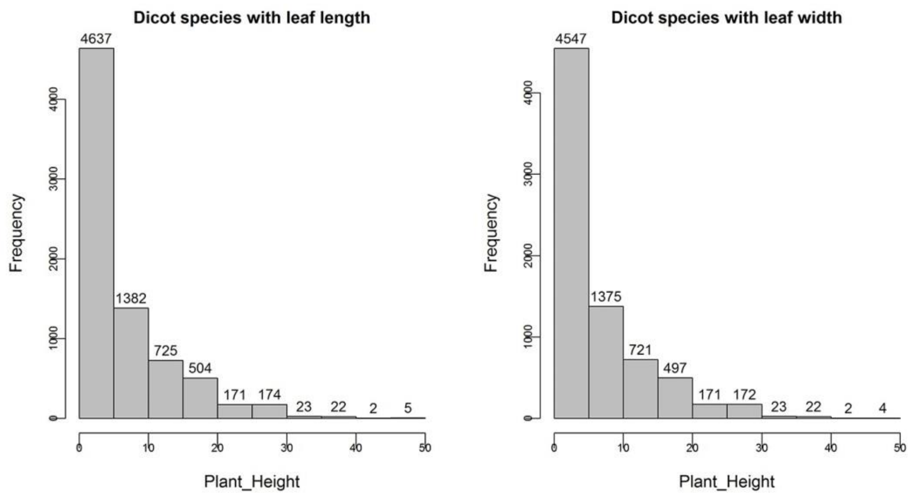

Figure S1.3 The frequency distributions of plant height for species with leaf size measures.

For each species with leaf length or width data (unit: cm), we collected their height data (unit: m) from *eflora*. The mean height of each species was calculated (species without erect or independent stems were excluded) (Wang *et al.* 2019). In total 7645 erect dicot species had both leaf length and height data; the height of 397 species exceeded 20 m and 1229 species had heights between 10 and 20 m. Similarly, 7534 erect dicot species had both leaf width and height data; here the height of 394 species exceeded 20 m and 1218 species had heights between 10 and 20 m.

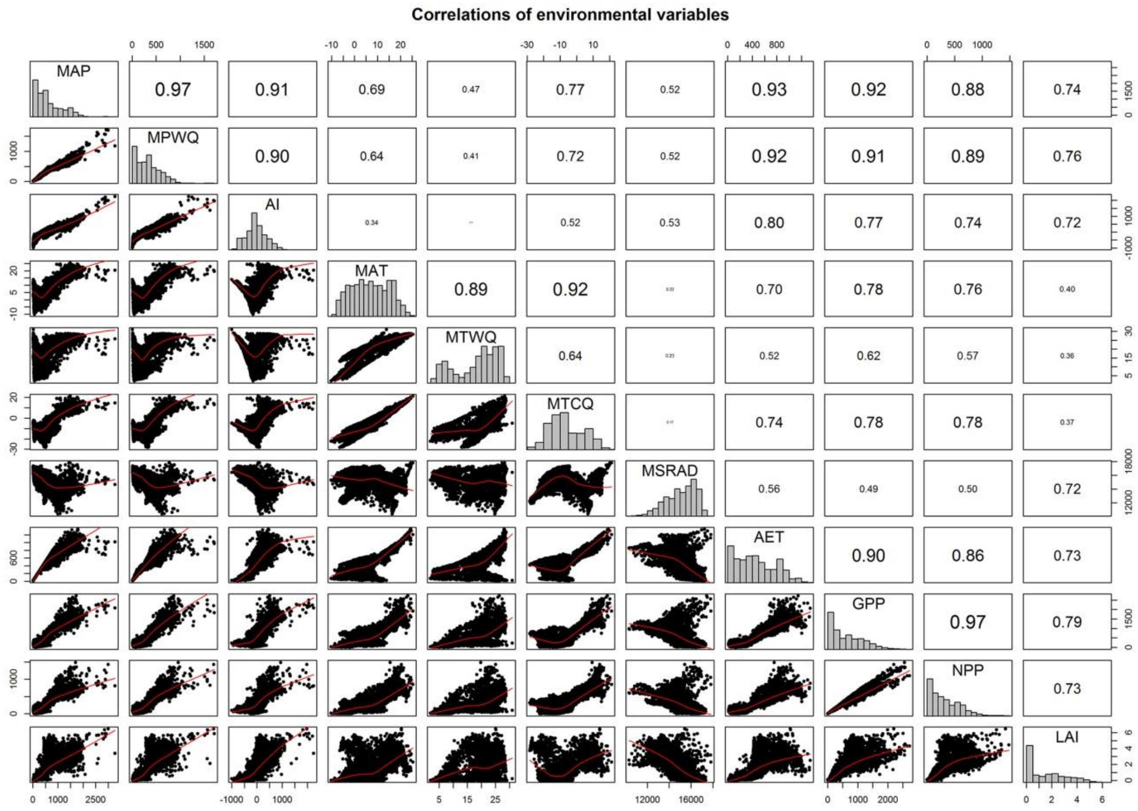

Figure S1.4 Correlation chart of environmental variables. The frequency distributions of gridded-mean environmental variables are shown in diagonal panels, and the titles represent the corresponding environmental variables from top-left to lower-right: mean annual precipitation (MAP, mm), precipitation of wettest quarter (MPWQ, mm), aridity index (AI, mm), mean annual temperature (MAT, ^{o}C), mean temperature of warmest quarter (MTWQ, ^{o}C), mean temperature of coldest quarter (MTCQ, ^{o}C), mean annual sun radiation (MSRAD, kJ cm^{-2} day^{-1}), annual actual evapotranspiration (AET, mm), gross primary productivity (GPP, gC $m^{-2}yr^{-1}$), net primary productivity (NPP, gC $m^{-2}yr^{-1}$) and mean leaf area index of July (LAI). GPP and NPP were derived from MODIS products. The corresponding correlation coefficients of each pair of environmental variables are shown in the upper right triangle and the font sizes correspond to the size of the values. Scatterplots with smooth lines (estimated by the LOESS method) are shown in the lower left triangle.

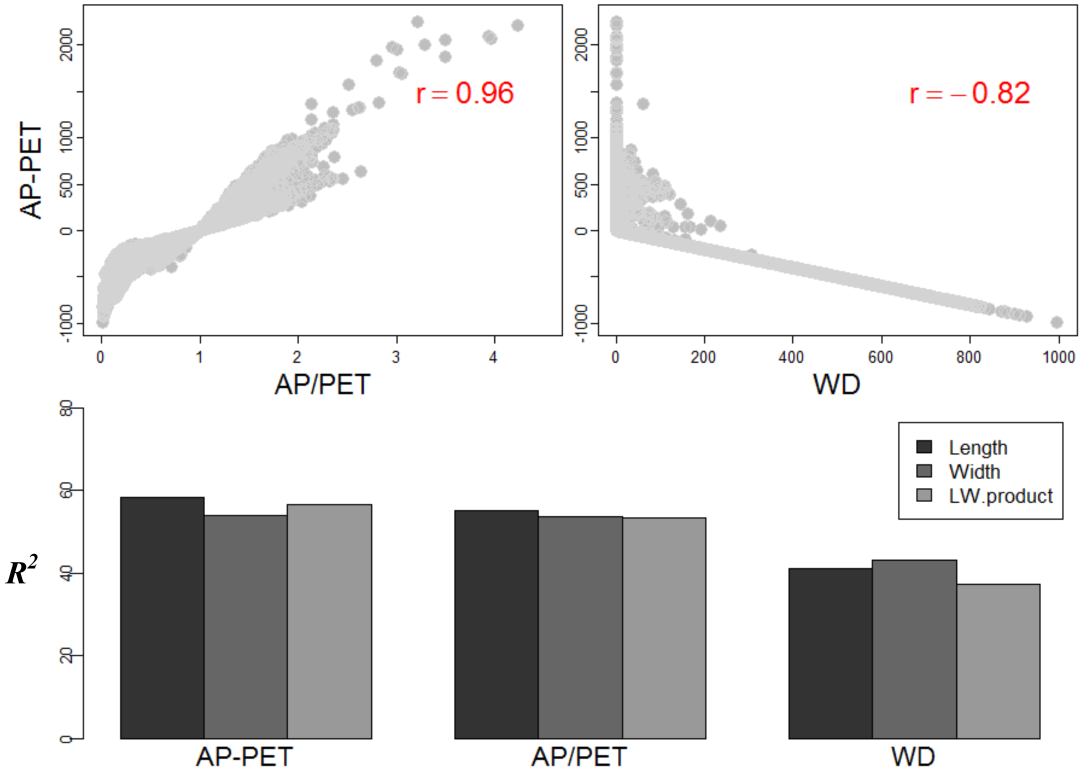

Figure S1.5 The correlations among three aridity indices (AP-PET, AP/PET and WD) and their relationship with the three measures of leaf size.

Note: In our preliminary analyses, we used water deficit (WD = PET–AET, mm) and two different definitions of AI, that is, AI = MAP/PET (United Nations Environment 1992) and AI = MAP–PET (Klein *et al.* 2015), to represent the aridity of a region. The two scatter plots in the first row show the correlations between the pairs of aridity indices (correlation coefficients shown in top right corner of panels). The three plots below show the relationships between leaf size measures and aridity indices. R-square values were extracted from the OLS linear models. The results showed that AI = MAP–PET was the best predictor of leaf size among these three variables. Therefore, only AI (= MAP–PET) was used in the analyses presented in the main text.

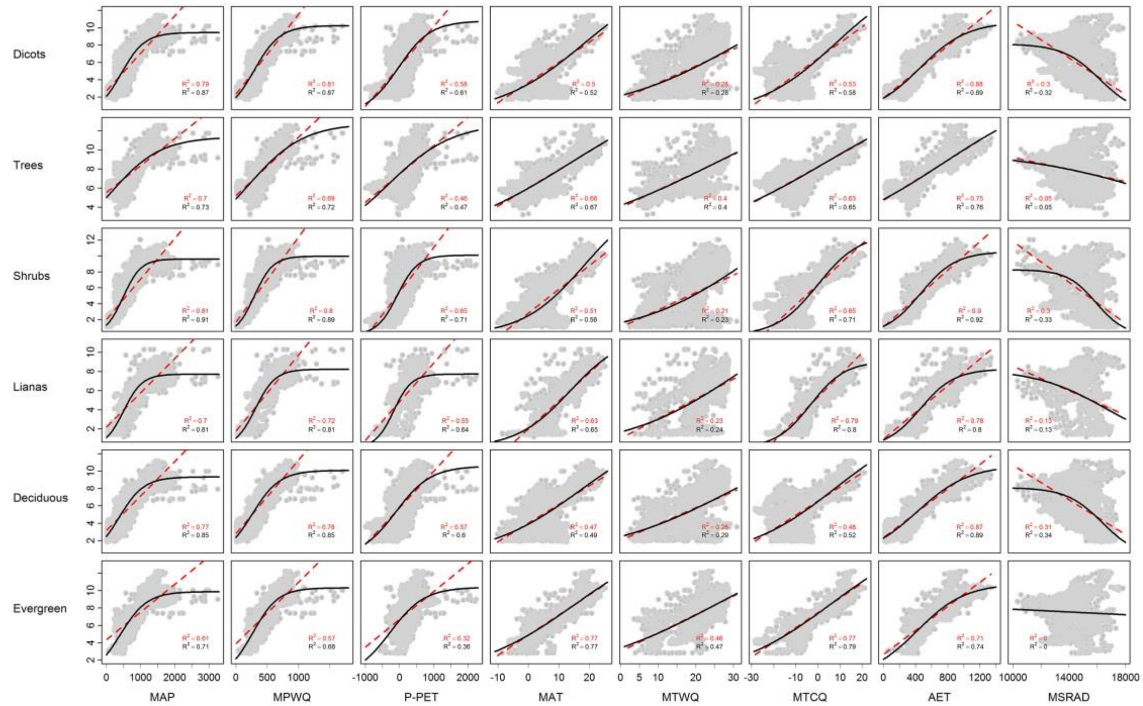

Figure S1.6 The relationships between environmental variables and average leaf length of woody dicots with different life forms. The vertical axes represent the average leaf length of woody dicots with different life forms (from top to bottom: all woody dicots, trees, shrubs, woody lianas, deciduous dicots, and evergreen dicots). Horizontal axes represent environmental variables (from left to right: mean annual precipitation (MAP, mm), precipitation of wettest quarter (MPWQ, mm), aridity index (AI, mm), mean annual temperature (MAT, ^{o}C), mean temperature of warmest quarter (MTWQ, ^{o}C), mean temperature of coldest quarter (MTCQ, ^{o}C), annual actual evapotranspiration (AET, mm) and annual sun radiation (SRAD, kJ cm^{-2} day^{-1})). Both logistic curves (black solid lines) and linear regressions (red dashed lines) were fitted to derive the relationships between the leaf size measures and environmental variables. Coefficients of determination (R^2) are shown in black for logistic curves and in red for linear regressions. Modified *t*-tests were used to calculate *p* values; no curves were drawn when $p > 0.05$. Each plot here is compressed into a dot in the first panel (a, leaf length) of Figure 1 in the main text.

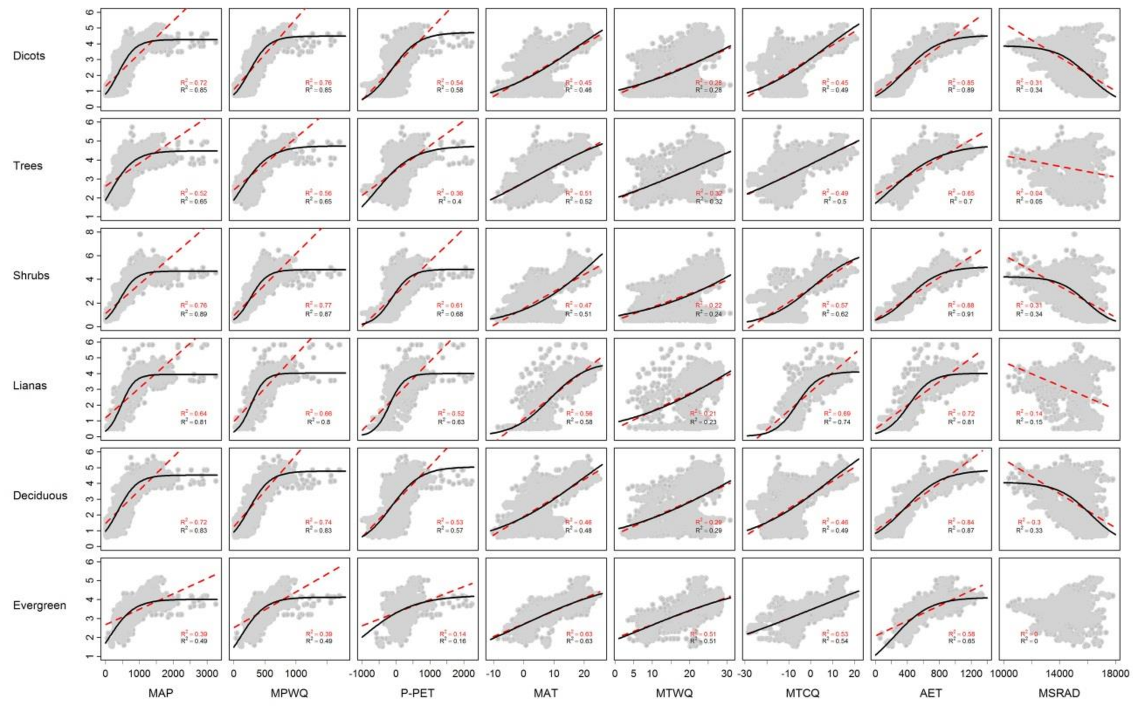

Figure S1.7 The relationships between environmental variables and average leaf width of woody dicots with different life forms. The meaning of vertical and horizontal axes is the same as in Figure S1.6. Each plot here is compressed into a dot in the second panel (b, leaf width) of Figure 1 in the main text.

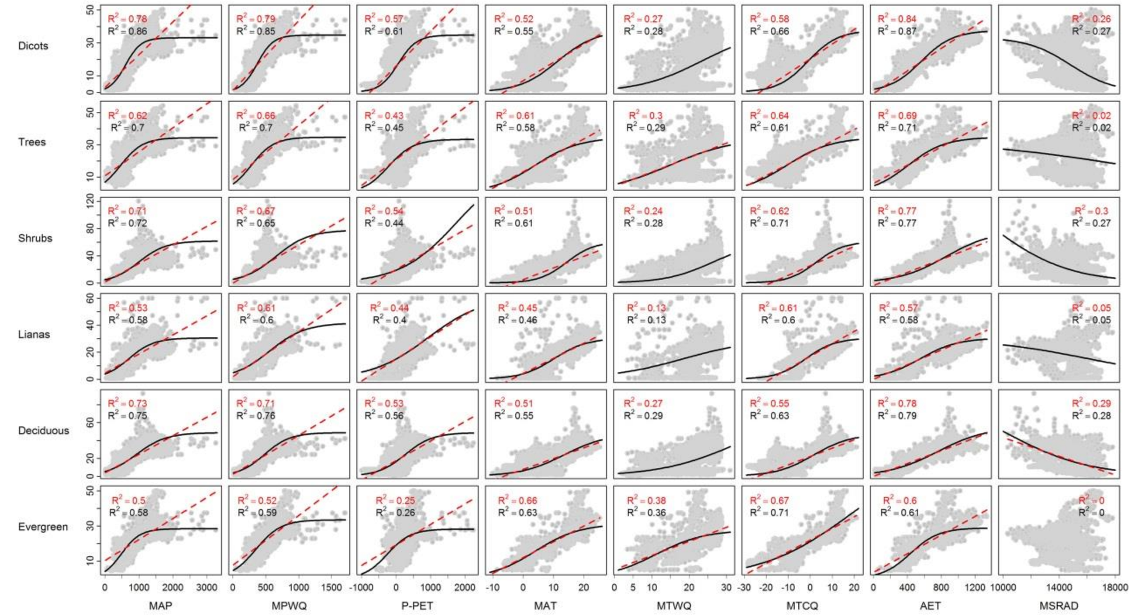

Figure S1.8 The relationships between environmental variables and average length-width product of leaves for woody dicots with different life forms. The meaning of vertical and horizontal axes is the same as in Figure S1.6. Each plot here is compressed into a dot in the third panel (c, leaf length-width product, with a corerction factor of 2/3) of Figure 1 in the main text.

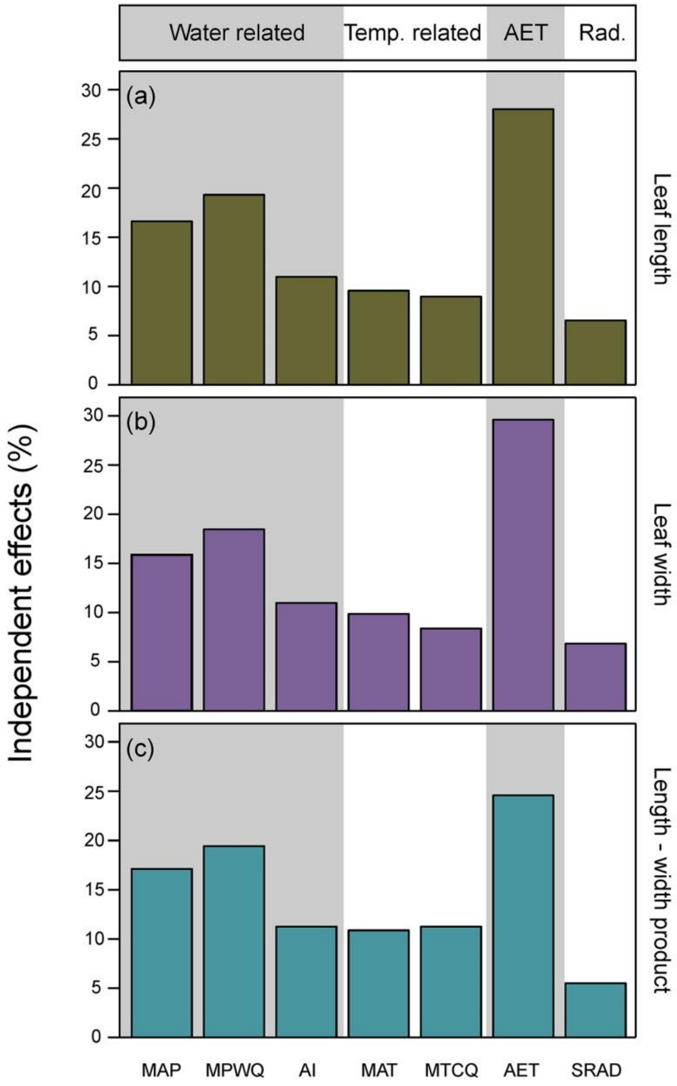

Figure S1.9 The independent effects of the environmental variables on leaf size evaluated with multiple hierarchical partitioning analyses. Leaf size of all woody dicots in China was measured by leaf length (a), leaf width (b) and leaf length-width product (c). See Figure 1 in the main text for the abbreviations of the environmental variables.

Table S1.1 Correlation coefficients between the gridded-mean leaf size measures based on the median and the maximum value of each species across different life forms.

(a) leaf length (LL, cm)

	**LL** $_{med}$	**LL_Trees** $_{med}$	**LL_Shrubs** $_{med}$	**LL_Lianas** $_{med}$	**LL_Deciduous** $_{med}$	**LL_Evergreen** $_{med}$
**LL** $_{max}$	1.00	0.96	0.99	0.97	0.99	0.98
**LL_Trees** $_{max}$	0.92	0.93	0.91	0.92	0.87	0.93
**LL_Shrubs** $_{max}$	0.98	0.95	0.97	0.95	0.97	0.96
**LL_Lianas** $_{max}$	0.78	0.78	0.72	0.83	0.73	0.80
**LL_Deciduous** $_{max}$	0.99	0.94	0.98	0.96	0.99	0.94
**LL_Evergreen** $_{max}$	0.96	0.94	0.92	0.90	0.92	0.97

(b) leaf width (LW, cm)

	**LW** $_{med}$	**LW_Trees** $_{med}$	**LW_Shrubs** $_{med}$	**LW_Lianas** $_{med}$	**LW_Deciduous** $_{med}$	**LW_Evergreen** $_{med}$
**LW** $_{max}$	1.00	0.95	0.98	0.96	0.99	0.93
**LW_Trees** $_{max}$	0.68	0.76	0.61	0.64	0.67	0.56
**LW_Shrubs** $_{max}$	0.98	0.90	0.98	0.93	0.97	0.89
**LW_Lianas** $_{max}$	0.43	0.55	0.33	0.47	0.31	0.55
**LW_Deciduous** $_{max}$	0.99	0.94	0.97	0.95	0.99	0.89
**LW_Evergreen** $_{max}$	0.92	0.79	0.86	0.77	0.86	0.90

(c) leaf length-width product (LWs, cm^2)

	**LWs** $_{med}$	**LWs_Trees** $_{med}$	**LWs_Shrubs** $_{med}$	**LWs_Lianas** $_{med}$	**LWs_Deciduous** $_{med}$	**LWs_Evergreen** $_{med}$
**LWs** $_{max}$	1.00	0.96	0.95	0.90	0.97	0.94
**LWs_Trees** $_{max}$	0.87	0.91	0.67	0.83	0.74	0.85
**LWs_Shrubs** $_{max}$	0.96	0.91	0.91	0.84	0.92	0.91
**LWs_Lianas** $_{max}$	0.44	0.56	0.14	0.62	0.17	0.58
**LWs_Deciduous** $_{max}$	0.98	0.95	0.92	0.87	0.95	0.91
**LWs_Evergreen** $_{max}$	0.81	0.60	0.82	0.55	0.79	0.68

Note: Both the median and maximum values of the three leaf size measures were used to calculate the average leaf length (LL), average leaf width (LW) and average length-width product (LWs) per grid cell. All these parameters were calculated separately for all woody dicots and different life forms (trees, shrubs, and woody lianas; deciduous and evergreen species). Then, the correlation coefficients were extracted to compare the influence of different leaf size measures (median vs. maximum).

Table S1.2 Correlation coefficients between the three leaf size measures of all woody dicots in China.

	Length	Width
**Width**	0.98	
**Length-width product**	0.98	0.97

Table S1.3 Correlation coefficients between different life forms of woody dicots for each leaf size measure. (a) leaf length (LL, cm); (b) leaf width (LW, cm); (c) leaf length-width product (LWs, cm^2).

(a) LL

LL	Dicots	Trees	Shrubs	Lianas	Deciduous	Evergreen
**Dicots**	1.00					
**Trees**	0.93	1.00				
**Shrubs**	0.97	0.87	1.00			
**Lianas**	0.93	0.88	0.89	1.00		
**Deciduous**	0.99	0.93	0.94	0.91	1.00	
**Evergreen**	0.91	0.86	0.89	0.84	0.88	1.00

(b) LW

LW	Dicots	Trees	Shrubs	Lianas	Deciduous	Evergreen
**Dicots**	1.00					
**Trees**	0.95	1.00				
**Shrubs**	0.96	0.87	1.00			
**Lianas**	0.91	0.83	0.85	1.00		
**Deciduous**	0.99	0.95	0.94	0.89	1.00	
**Evergreen**	0.78	0.72	0.77	0.68	0.73	1.00

(c) LWs

LWs	Dicots	Trees	Shrubs	Lianas	Deciduous	Evergreen
**Dicots**	1.00					
**Trees**	0.96	1.00				
**Shrubs**	0.94	0.85	1.00			
**Lianas**	0.80	0.74	0.66	1.00		
**Deciduous**	0.97	0.91	0.95	0.75	1.00	
**Evergreen**	0.81	0.79	0.73	0.59	0.73	1.00

Table S1.4 Summary of the numbers of grid cells included in the analyses for species with different life forms. Grid cells with less than 20 species were excluded in the regression analyses. The three columns show the numbers of grid cells included in the regression, excluded in the regressions and the proportion of excluded grid cells.

Leaf size	Life forms	# of grid cells for regression	# of grid cells with less than 20 species	Proportion excluded
Leaf Length	**Dicots**	3522	272	0.072
	**Trees**	2697	1097	0.289
	**Shrubs**	2990	804	0.212
	**Lianas**	1819	1975	0.521
	**Deciduous**	3427	367	0.097
	**Evergreen**	1911	1883	0.496
Leaf width	**Dicots**	3521	273	0.072
	**Trees**	2664	1130	0.298
	**Shrubs**	2948	846	0.223
	**Lianas**	1809	1985	0.523
	**Deciduous**	3384	410	0.108
	**Evergreen**	1910	1884	0.497
Length-width product	**Dicots**	3521	273	0.072
	**Trees**	2659	1135	0.299
	**Shrubs**	2948	846	0.223
	**Lianas**	1804	1990	0.525
	**Deciduous**	3384	410	0.108
	**Evergreen**	1910	1884	0.497

Table S1.5 R^2 -values of the relationships between environmental variables and leaf size measures for woody dicots and different life forms obtained from logistic curves. *P* values were calculated with a modified *t*-test.

Life forms		MAP		MPWQ		AI		MAT		MTWQ		MTCQ		AET		SRAD	
(Woody dicots)		R^2	*p*	R^2	*p*	R^2	*p*	R^2	*p*	R^2	*p*	R^2	*p*	R^2	*P*	R^2	*P*
**Leaf Length**	**Dicots**	0.87	***	0.87	***	0.61	***	0.52	***	0.28	***	0.58	***	0.89	***	0.32	***
	**Trees**	0.73	***	0.72	***	0.47	***	0.67	***	0.40	***	0.65	***	0.76	***	0.05	**
	**Shrubs**	0.91	***	0.89	***	0.71	***	0.56	***	0.23	***	0.71	***	0.92	***	0.33	***
	**Lianas**	0.81	***	0.81	***	0.64	***	0.65	***	0.24	***	0.80	***	0.80	***	0.13	**
	**Deciduous**	0.85	***	0.85	***	0.60	***	0.49	***	0.29	***	0.52	***	0.89	***	0.34	***
	**Evergreen**	0.71	***	0.68	***	0.36	***	0.77	***	0.47	***	0.79	***	0.74	***	0.00	
**Leaf Width**	**Dicots**	0.85	***	0.85	***	0.58	***	0.46	***	0.28	***	0.49	***	0.89	***	0.34	***
	**Trees**	0.65	***	0.65	***	0.40	***	0.52	***	0.32	***	0.50	***	0.70	***	0.05	**
	**Shrubs**	0.89	***	0.87	***	0.68	***	0.51	***	0.24	***	0.62	***	0.91	***	0.34	***
	**Lianas**	0.81	***	0.80	***	0.63	***	0.58	***	0.23	***	0.74	***	0.81	***	0.15	**
	**Deciduous**	0.83	***	0.83	***	0.57	***	0.48	***	0.29	***	0.49	***	0.87	***	0.33	***
	**Evergreen**	0.49	***	0.49	***	0.16	***	0.63	***	0.51	***	0.54	***	0.65	***	0.00	
**Length-width product**	**Dicots**	0.86	***	0.85	***	0.61	***	0.58	***	0.29	***	0.67	***	0.87	***	0.28	***
	**Trees**	0.70	***	0.71	***	0.46	***	0.62	***	0.31	***	0.67	***	0.72	***	0.02	*
	**Shrubs**	0.73	***	0.71	***	0.57	***	0.61	***	0.29	***	0.71	***	0.80	***	0.29	***
	**Lianas**	0.58	***	0.63	***	0.48	***	0.46	***	0.13	***	0.60	***	0.58	***	0.05	*
	**Deciduous**	0.76	***	0.77	***	0.56	***	0.56	***	0.29	***	0.63	***	0.81	***	0.29	***
	**Evergreen**	0.58	***	0.59	***	0.27	***	0.67	***	0.38	***	0.71	***	0.62	***	0.00	

Note: *** $p < 0.001$; ** $p < 0.01$; * $p < 0.05$.

Table S1.6 R^2-values of the relationships between environmental factors and leaf size measures for woody dicots and different life forms obtained from linear regressions. *P* values were calculated with a modified *t* test.

Life forms (Woody dicots)		MAP		MPWQ		AI		MAT		MTWQ		MTCQ		AET		SRAD	
		R^2	*p*	R^2	*p*	R^2	*p*	R^2	*p*	R^2	*p*	R^2	*p*	R^2	*P*	R^2	*P*
**Leaf Length**	**Dicots**	0.79	***	0.81	***	0.58	***	0.50	***	0.28	***	0.53	***	0.88	***	0.30	***
	**Trees**	0.70	***	0.69	***	0.46	***	0.66	***	0.40	***	0.63	***	0.75	***	0.05	**
	**Shrubs**	0.81	***	0.80	***	0.65	***	0.51	***	0.21	***	0.65	***	0.90	***	0.30	***
	**Lianas**	0.70	***	0.72	***	0.55	***	0.63	***	0.23	***	0.79	***	0.78	***	0.13	**
	**Deciduou**	0.77	***	0.78	***	0.57	***	0.47	***	0.28	***	0.48	***	0.87	***	0.31	***
	**Evergreen**	0.61	***	0.57	***	0.32	***	0.77	***	0.46	***	0.77	***	0.71	***	0.00	
**Leaf Width**	**Dicots**	0.72	***	0.76	***	0.54	***	0.45	***	0.28	***	0.45	***	0.85	***	0.31	***
	**Trees**	0.52	***	0.56	***	0.36	***	0.51	***	0.32	***	0.49	***	0.65	***	0.04	**
	**Shrubs**	0.76	***	0.77	***	0.61	***	0.47	***	0.22	***	0.57	***	0.88	***	0.31	***
	**Lianas**	0.64	***	0.66	***	0.52	***	0.56	***	0.21	***	0.69	***	0.72	***	0.14	**
	**Deciduou**	0.72	***	0.74	***	0.53	***	0.46	***	0.29	***	0.46	***	0.84	***	0.30	***
	**Evergreen**	0.39	***	0.39	***	0.14	***	0.63	***	0.51	***	0.53	***	0.58	***	0.00	
**Length-width product**	**Dicots**	0.78	***	0.79	***	0.57	***	0.52	***	0.27	***	0.58	***	0.84	***	0.26	***
	**Trees**	0.62	***	0.66	***	0.43	***	0.61	***	0.30	***	0.64	***	0.69	***	0.02	*
	**Shrubs**	0.71	***	0.67	***	0.54	***	0.51	***	0.24	***	0.62	***	0.77	***	0.30	***
	**Lianas**	0.53	***	0.61	***	0.44	***	0.45	***	0.13	***	0.61	***	0.57	***	0.05	*
	**Deciduou**	0.73	***	0.71	***	0.53	***	0.51	***	0.27	***	0.55	***	0.78	***	0.29	***
	**Evergreen**	0.50	***	0.52	***	0.25	***	0.66	***	0.38	***	0.67	***	0.60	***	0.00	

Note: *** $p < 0.001$; ** $p < 0.01$; * $p < 0.05$.

Appendix 2 **The interactive effects of energy and water on average leaf size.**

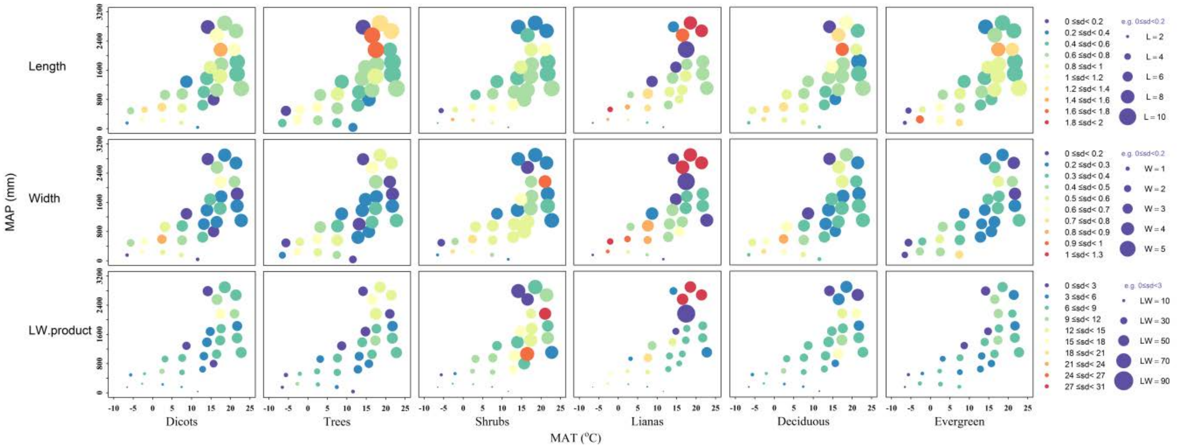

Figure S2.1 The interactive effects of mean annual temperature (MAT) and annual precipitation (MAP) on leaf size measures for all woody dicots and different life forms. The three rows of plots represent the three leaf size measures and the six columns represent the six different life forms of woody plants (see labels). The size of dots indicates the mean value of leaf size measures while the color of the dots indicates the corresponding standard deviation within grid cells at given temperature and precipitation bins (2 ℃ for MAT and 200 mm for MAP).

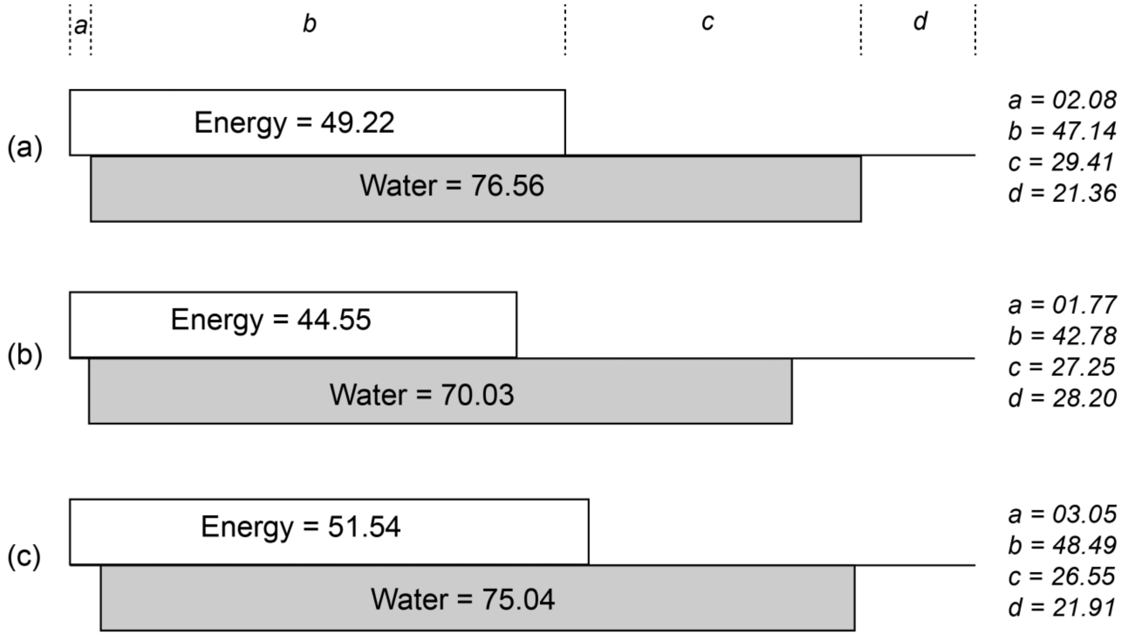

Figure S2.2 Comparison between the effects of energy and water on leaf size measures of all woody dicots based on partial regression. "Energy" is the first axis derived form a principal component analysis (PCA) with three temperature variables (MAT, MTCQ, MTWQ), and "Water" is the first axis derived from a PCA with three precipitation variables (MAP, MPDQ, MPWQ). Both axes represent more than 87% variation. (a) leaf length; (b) leaf width; (c) leaf length-width product. The variation of corresponding leaf size measures is partitioned into four parts: a, independent component of energy; b, shared component where the influence of energy and water are confounded and therefore cannot be separated; c, independent component of water; d, residual variation.

Note: We conducted variance partitioning analysis to compare the relative influences of energy and water on leaf size measures of woody plants in China, where energy and water variables are, in general, strongly correlated with each other. Firstly, we chose mean annual temperature (MAT), mean temperature of coldest quarter (MTCQ) and mean temperature of warmest quarter (MTWQ) to represent environmental energy, and mean annual precipitation (MAP), precipitation of wettest quarter (MPWQ) and precipitation of driest quarter (MPDQ) to represent water availability. Secondly, we conducted PCA on energy and water variables separately, and extracted the first principal components for energy (PCAe) and water (PCAw). Both PCAe and PCAw explained over 87% of the total variance of energy and water variables respectively. Thirdly, three linear models were built, including PCAe, PCAw or both as predictor variables. Using partial regression, we assessed the independent and joint contributions of energy and water in explaining the spatial variations of leaf size measures.

Appendix 3 **The influence of evolutionary age on the relationship between leaf size and primary productivity.**

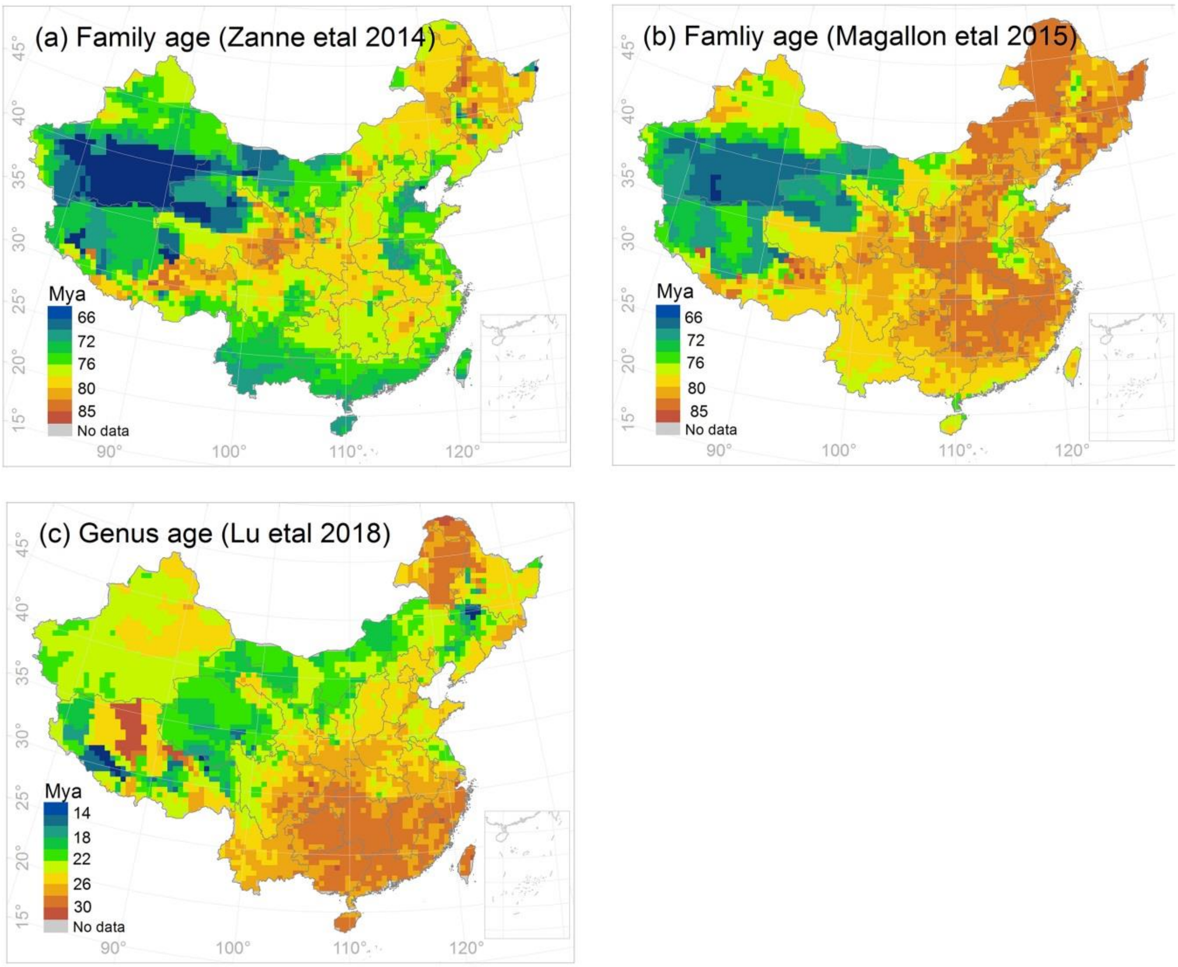

Figure S3.1 The geographical patterns in average family age and genus age of all woody dicot species in China. a), family age (Mya) calculated from the family-level phylogeny constructed by Zanne *et al.* (2014); b), family age (Mya) calculated from the family-level phylogeny constructed by Magallon *et al.* (2015); c), genus age (Mya) calculated from the genus-level phylogeny constructed by Lu *et al.* (2018). Totally 3,794 grid cells with a spatial resolution of 50 × 50 km are shown. Values increase from blue to red.

Table S3.1 Phylogenetic signals for the mean values of leaf size within family and genus. Two dated family-level phylogenies constructed by Zanne *et al.* (2014) and Magallon *et al.* (2015) and one dated genus-level phylogeny from Lu *et al.* (2018) were used. The significant values are in bold face.

**Phylogenies**		**Family level Zanne's tree**		**Family level Magallon's tree**		**Genus level Lu's tree**	
**Phylogenetic Signal**		**K**	***p***	**K**	***p***	**K**	***p***
**Leaf length**	**Dicots**	0.598	0.164	**0.843**	**0.036**	**0.149**	**0.003**
	**Trees**	0.560	0.608	0.810	0.382	0.140	0.092
	**Shrubs**	**0.845**	**0.015**	**1.119**	**0.000**	0.090	0.766
	**Lianas**	0.615	0.484	0.918	0.200	**0.244**	**0.006**
	**Deciduous**	0.609	0.166	0.784	0.393	0.250	0.123
	**Evergreen**	0.511	0.747	0.732	0.727	0.168	0.071
**Leaf width**	**Dicots**	0.568	0.268	0.831	0.059	0.116	0.417
	**Trees**	0.565	0.591	0.808	0.417	0.106	0.636
	**Shrubs**	0.620	0.142	**0.931**	**0.012**	0.091	0.818
	**Lianas**	0.614	0.491	0.895	0.330	**0.252**	**0.011**
	**Deciduous**	0.550	0.636	0.801	0.353	**0.272**	**0.011**
	**Evergreen**	0.535	0.626	0.756	0.597	0.113	0.684

Note: To estimate phylogenetic signals of leaf size, we first calculated the mean value of leaf length and width for each family/genus of Chinese woody dicots, and matched them with the three phylogenies separately (Zanne *et al.* 2014; Magallon *et al.* 2015; Lu *et al.* 2018). Then Blomberg's *K* (Blomberg *et al.* 2003) were used to estimate the phylogenetic signals for leaf size of all dicots, and those of trees, shrubs and lianas separately.

Table S3.2 The influence of clade age on the relationships between leaf size and annual actual evapotranspiration (AET). LL, community mean leaf length; LW, leaf width; LW.prod, leaf length-width production per grid cell of all woody dicots. Fage_Zanne, mean family age per grid cell based on the phylogeny of Zanne *et al.* (2014); Fage_Magallon, mean family age based on the phylogeny of Magallon *et al.* (2015); Fage_Lu, mean genus age per grid cell based on the phylogeny of Lu *et al.* (2018).. %SS, the proportion of sum of squares of the corresponding explained variable (this corresponds to R^2 * 100); Coefficient, the regression coefficient (cm/mm). Here, both LL and LW were sqrt-transformed considering their nonlinear relationships with AET. Note that despite the small effects of family age and the interaction these are often highly significant due to the large sample size. As discussed in the main text, the mostly negative regression coefficients for the interactions demonstrate that grid cells in which species belong to older families show weaker correlations between leaf size and climate, indicating that there the species show slightly stronger evolutionary constraints regarding leaf size adaptations to contemporary climates.

a)

Response variable	Explanatory variables	%SS	Significance	Coefficient
LL	AET	86.936	$p < 0.001$	0.0017
	Fage_Zanne	0.402	$p < 0.001$	0.0087
	AET: Fage_Zanne	0.001	$p > 0.05$	-0.0000013
LW	AET	83.061	$p < 0.001$	0.00034
	Fage_Zanne	1.960	$p < 0.001$	0.011
	AET: Fage_Zanne	0.075	$p < 0.001$	0.000011
LW.prod	AET	85.152	$p < 0.001$	0.0025
	Fage_Zanne	0.095	$p < 0.001$	0.0036
	AET: Fage_Zanne	0.037	$p < 0.01$	0.000028

b)

Response variable	Explanatory variables	%SS	Significance	Coefficient
LL	AET	86.936	$p < 0.001$	0.00078
	Fage_Magallon	0.853	$p < 0.001$	0.012
	AET: Fage_Magallon	0.024	$p < 0.05$	0.0000095
LW	AET	83.061	$p < 0.001$	-0.00043
	Fage_Magallon	3.505	$p < 0.001$	0.018
	AET: Fage_Magallon	0.160	$p < 0.001$	0.000018
LW.prod	AET	85.1512	$p < 0.001$	-0.00093
	Fage_Magallon	0.466	$p < 0.001$	0.016
	AET: Fage_Magallon	0.149	$p < 0.001$	0.000067

c)

Response variable	Explanatory variables	%SS	Significance	Coefficient
LL	AET	86.936	$p < 0.001$	0.0035
	Gage_Lu	1.68	$p < 0.001$	0.070
	AET: Gage_Lu	1.116	$p < 0.001$	-0.000083
LW	AET	83.061	$p < 0.001$	0.0041
	Gage_Lu	1.513	$p < 0.001$	0.078
	AET: Gage_Lu	4.307	$p < 0.001$	-0.00012
LW.prod	AET	85.152	$p < 0.001$	0.010
	Gage_Lu	1.407	$p < 0.001$	0.19
	AET: Gage_Lu	1.186	$p < 0.001$	-0.00024

Table S3.3 The influence of mean family age on the relationships between leaf size and primary productivity. a-b) Fage_Zanne, mean family age per grid cell based on the phylogeny of Zanne *et al.* (2014); **c-d)** Fage_Magallon, grid-mean family age based on the phylogeny of Magallon *et al.* (2015). %SS, the proportion of sum of squares of the corresponding explained variable (this corresponds to $R^2 * 100$); Coefficient, the regression coefficient (gC $m^{-2}yr^{-1}$/cm). Here, both NPP and GPP were sqrt-transformed considering their nonlinear relationships with leaf size.

a)

**Response variable**	**Explanatory variables**	**%SS**	**Significance**	**Coefficient**
NPP	LL	81.787	$p < 0.001$	5.99
	Fage_Zanne	0.003	$p > 0.05$	0.175
	LL: Fage_Zanne	0.315	$p < 0.001$	-0.046
GPP	LL	85.521	$p < 0.001$	4.871
	Fage_Zanne	0	$p > 0.05$	0.047
	LL: Fage_Zanne	0.012	$p > 0.05$	-0.013

b)

**Response variable**	**Explanatory variables**	**%SS**	**Significance**	**Coefficient**
NPP	LW	79.689	$p < 0.001$	18.319
	Fage_Zanne	0.515	$p < 0.001$	0.198
	LW: Fage_Zanne	1.239	$p < 0.001$	-0.175
GPP	LW	85.53	$p < 0.001$	19.508
	Fage_Zanne	0.49	$p < 0.001$	0.103
	LW: Fage_Zanne	0.446	$p < 0.001$	-0.155

c)

**Response variable**	**Explanatory variables**	**%SS**	**Significance**	**Coefficient**
NPP	LL	81.787	$p < 0.001$	5.59
	Fage_Magallon	0.031	$p < 0.05$	0.172
	LL: Fage_Magallon	0.136	$p < 0.001$	-0.038
GPP	LL	85.521	$p < 0.001$	0.824
	Fage_Magallon	0.103	$p < 0.001$	-0.035
	LL: Fage_Magallon	0.061	$p < 0.001$	0.038

d)

**Response variable**	**Explanatory variables**	**%SS**	**Significance**	**Coefficient**
NPP	LW	79.689	$p < 0.001$	20.789
	Fage_Magallon	0.338	$p < 0.001$	0.178
	LW: Fage_Magallon	0.966	$p < 0.001$	-0.194
GPP	LW	83.53	$p < 0.001$	16.415
	Fage_Magallon	0.204	$p < 0.001$	0.019
	LW: Fage_Magallon	0.131	$p < 0.001$	-0.106

Table S3.4 The influence of mean genus age on the relationships between leaf size and primary productivity. Fage_Lu, mean genus age per grid cell based on the phylogeny of Lu *et al.* (2018). %SS, the proportion of sum of squares of the corresponding explained variable (this corresponds to R^2 * 100); Coefficient, the regression coefficient (gC $m^{-2}yr^{-1}$/cm). Here, both NPP and GPP were sqrt-transformed considering their nonlinear relationships with leaf size.

a)

Response variable	Explanatory variables	%SS	Significance	Coefficient
NPP	LL	81.787	p < 0.001	2.8
	Gage_Lu	0.238	p < 0.001	0.246
	LL: Gage_Lu	0.015	p > 0.05	-0.015
GPP	LL	85.52	p < 0.001	2.94
	Fage_Zanne	0.885	p < 0.001	0.39
	LL: Gage_Lu	0.014	p > 0.05	0.021

b)

Response variable	Explanatory variables	%SS	Significance	Coefficient
NPP	LW	79.689	p < 0.001	0.27
	Gage_Lu	0.724	p < 0.001	-0.161
	LW: Gage_Lu	0.563	p < 0.001	0.178
GPP	LW	83.53	p < 0.001	-1.677
	Gage_Lu	1.655	p < 0.001	-0.213
	LW: Gage_Lu	0.94	p < 0.001	0.339

Appendix 4 **The link of leaf size with ecosystem primary productivity.**

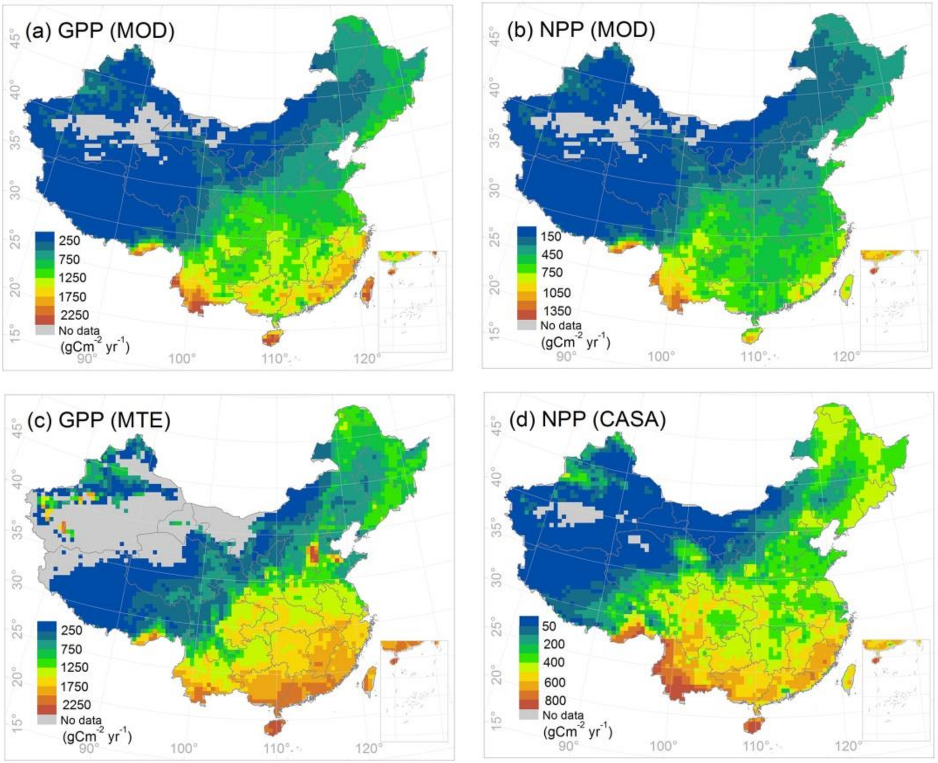

Figure S4.1 The geographical patterns of ecosystem primary productivity in China. a), MODIS-derived GPP (gross primary productivity, gC m^{-2}yr^{-1}); b), MODIS-derived NPP (net primary productivity, gC m^{-2}yr^{-1}); c), Flux-based GPP derived from the model tree ensemble (MTE) algorithm (gC m^{-2}yr^{-1}); and d), NPP calculated by the Carnegie-Ames-Stanford Approach (CASA) (gC m^{-2}yr^{-1}). From blue to red, the values increase. Note: The spatial resolution is 50 × 50 km. Correlations between MODIS- and flux-based GPP r=0.908, between MODIS-derived NPP and CASA-calculated NPP r=0.907.

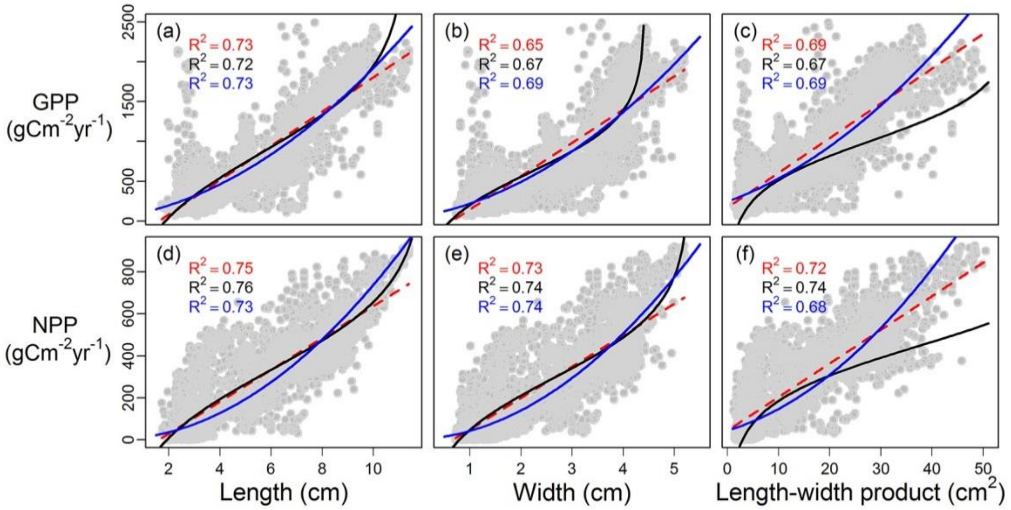

Figure S4.2 Relationships between average leaf size and ecosystem primary productivity in China. a-c), Flux-based GPP derived from the Model Tree Ensemble algorithm (gC $m^{-2}yr^{-1}$); d-f) and NPP calculated by the Carnegie-Ames-Stanford approach (gC $m^{-2}yr^{-1}$), respectively (see legend to Figure 2 for meaning of axes and Methods for details).

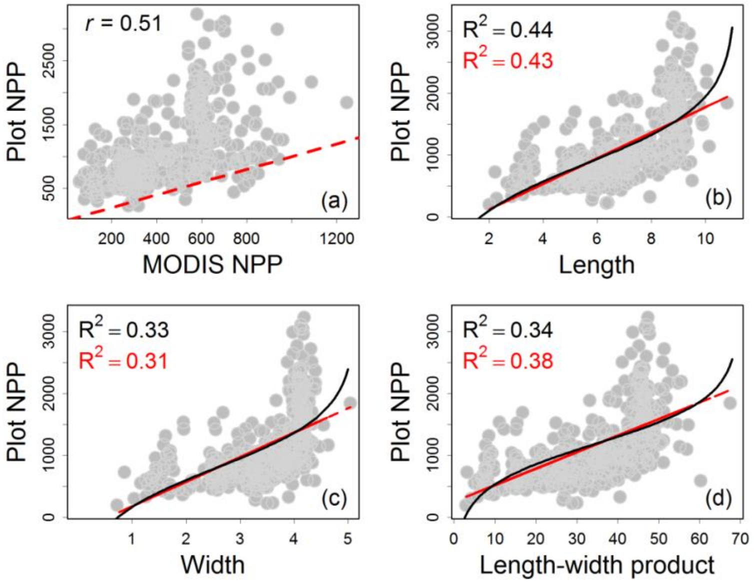

Figure S4.3 Relationships between average leaf size and net primary productivity (NPP) estimated based on forest plots. (a) Comparison of MODIS-derived NPP data (MODIS NPP, gC $m^{-2}yr^{-1}$) and those estimated from plot data about forest biomass (Plot NPP, gC $m^{-2}yr^{-1}$); (b-d) Relationship of Plot NPP with leaf length (b), leaf width (c), and leaf length-width product (d). For plot a, the red line shows the 1:1 reference line. For plots b-d, both results from linear regression (red) and inverse-logistic regression (black) are shown together with the corresponding R^2-values. All relationships were significant (p < 0.05).

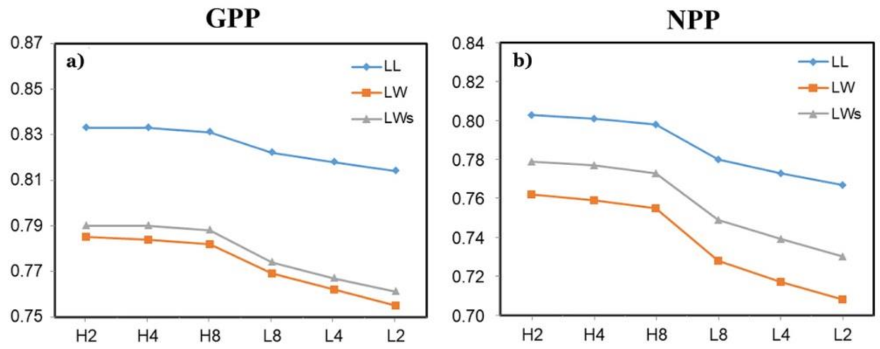

Figure S4.4 Relationships between leaf size and primary productivity among years with high or low values of primary productivity. a) GPP, MODIS-derived gross primary productivity; b) NPP, MODIS-derived net primary productivity. LL, mean leaf length; LW, mean leaf width; LWs, mean leaf length-width product. Vertical axes represent the coefficients of determination (R^2) of nonlinear regressions. H2, mean GPP and NPP per grid cell of the highest 2 years from 2000 to 2015; H4, those of the highest 4 years; H8, highest 8 years; L8, lowest 8 years; L4, lowest 4 years; L2, lowest 2 years. GPP and NPP decreased from H2 to L2. See Table S4.2 for exact R^2-values and those of the linear regressions.

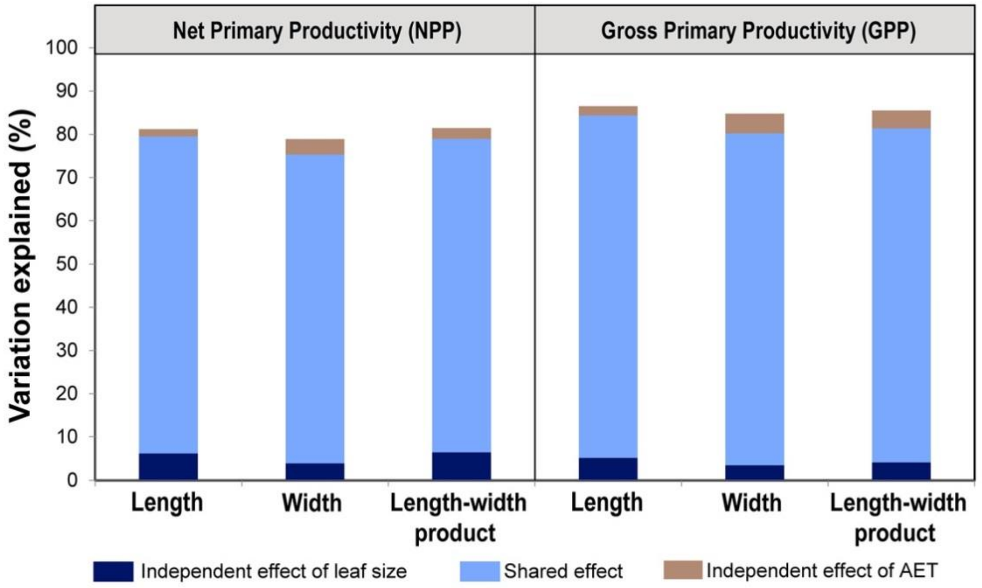

Figure S4.5 The independent and shared effects of leaf size and annual actual evapotranspiration (AET) on ecosystem primary productivity evaluated with partial regressions. The explained variations in productivity (i.e., MODIS-derived NPP and GPP) by each of the three leaf size measures (i.e., leaf length, leaf width and length-width product) and AET was partitioned into three parts: the independent effects of leaf size (dark blue) and AET (brown), and the shared effect (light blue). See Table S4.3 for the corresponding statistics of the partial regressions.

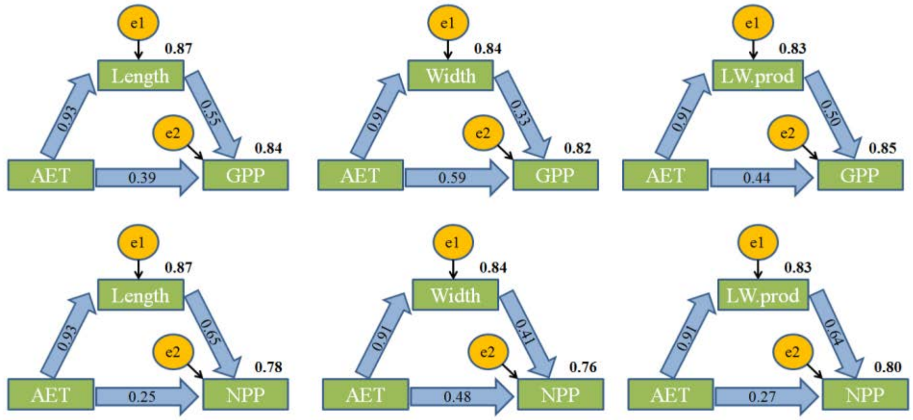

Figure S4.6 Relationships bewteen AET, primary productivity variables and leaf size measures based on standardized structural equation models (SEMs). Length, mean leaf length; Width, mean leaf width; LW.prod, mean leaf length-width product, AET, annual actual evapotranspiration; GPP, MOPDIS-derived gross primary productivity; NPP, MODIS-derived net primary productivity. The standardized path coefficients are shown in the arrows, and the coefficient of determination are shown in bold on the top right of the corresponding response variables.

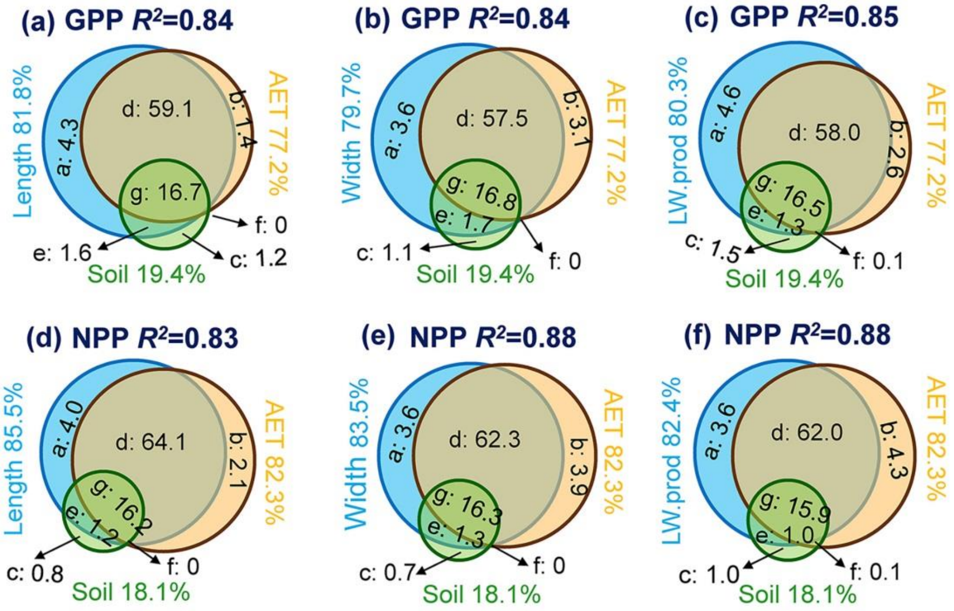

Figure S4.7 Venn diagrams to show the relative contribution of leaf size, AET and soil to variation in primary productivity variation in China. AET, annual actual evapotranspiration; Length, mean leaf length; Width, mean leaf width; LW.prod, mean leaf length-width product; Soil, soil characteristics; GPP, gross primary productivity; NPP, net primary productivity. Three soil variables (i.e. soil pH, soil organic carbon content and soil cation exchange capacity) were used to conduct Principal Component Analysis (PCA), and the first principal component, explaining > 70% of the total variation of the three soil variables, was extracted to characterize edaphic variation (soil characteristics). Both GPP and NPP were sqrt-transformed. The numbers in VDs show the explained percentage of total variation: the independent effect of leaf size (a), AET (b) and edaphic variation (c), and the joint effect of leaf size and AET (d), leaf size and edaphic variation (e), AET and edaphic variation (f), and all the three variables (g). The numbers around the circles show the total effect of leaf size (in blue), AET (in yellow) and soil characteristics (in green). The circle sizes are scaled by the R^2 of the corresponding regression.

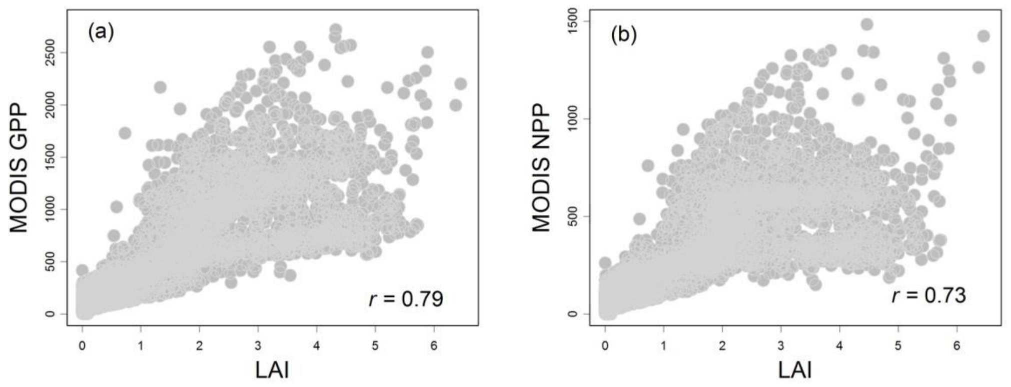

Figure S4.8 Relationships between leaf area index (LAI) and MODIS-derived primary productivity. (a) Correlation between MODIS-derived GPP and LAI; (b) Correlation between MODIS-derived NPP and LAI. The corresponding correlation coefficients are shown at the bottom right.

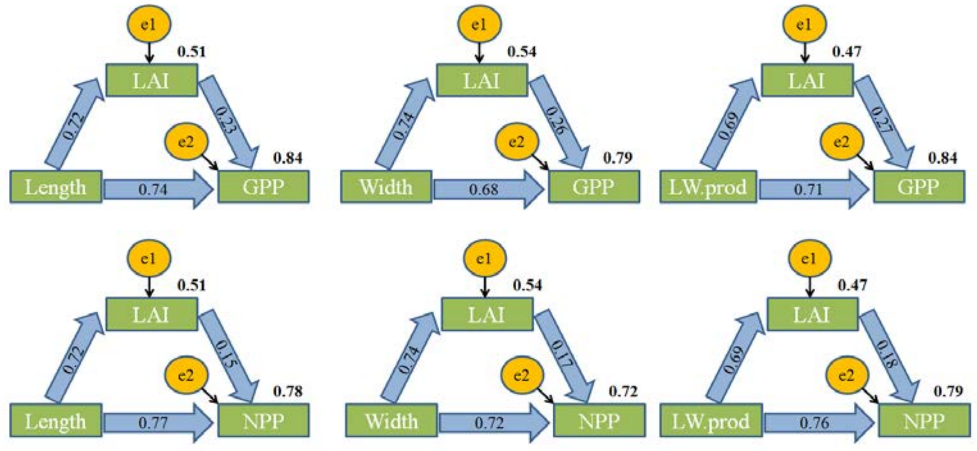

Figure S4.9 Relationships among leaf size, leaf area index (LAI) and primary productivity variables based on SEMs. See Figure S4.7 for the meaning of the captions and path coefficients. The results indicated that leaf size might mainly affect primary productivity directly rather than indirectly through its effect on LAI.

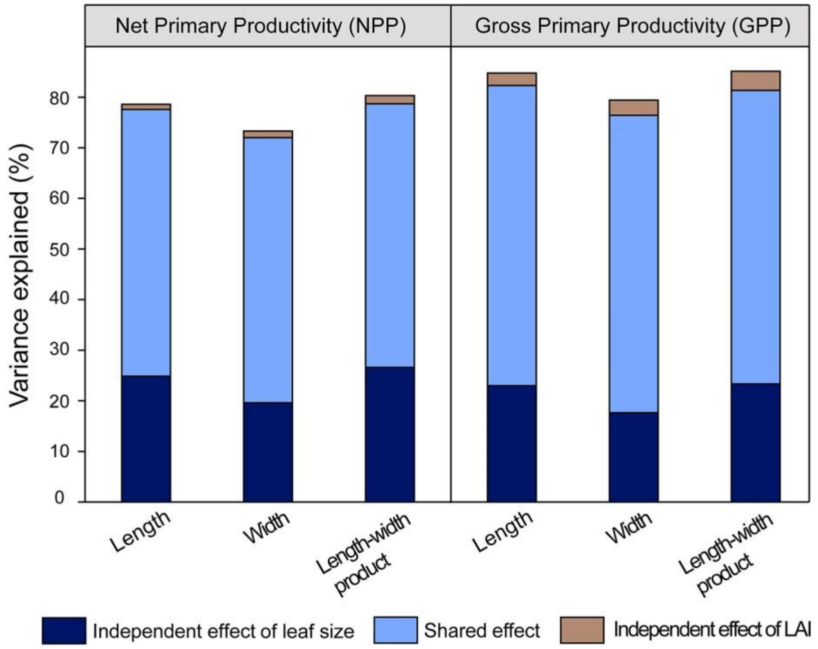

Figure S4.10 The independent and shared effects of leaf size and leaf area index (LAI) on ecosystem primary productivity evaluated with partial regressions. The explained variations in MODIS-derived NPP and GPP by each leaf size measure (i.e., leaf length, leaf width and length-width product) and LAI was partitioned into three parts: the independent effects of leaf size (dark blue) and LAI (brown), and the shared effect of the two (light blue).

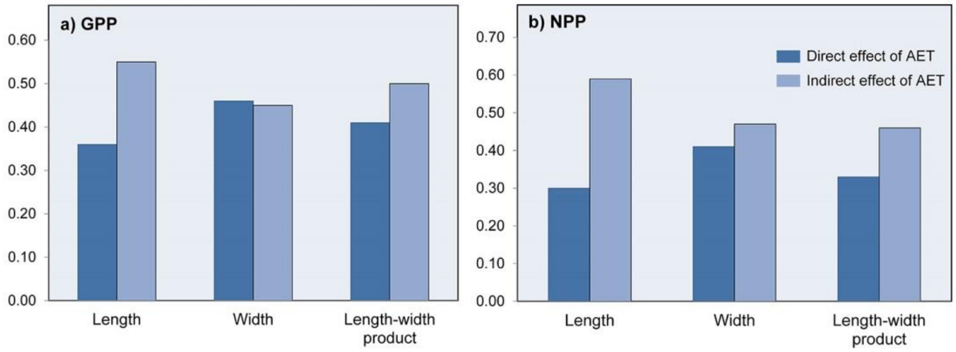

Figure S4.11 The direct and indirect effect of annual actual evapotranspiration (AET) on variation of primary production. The direct effect of AET to GPP and NPP and the indirect effect of AET via leaf size and leaf area index (LAI) to GPP and NPP were estimated based on the results of SEMs (See Figure 3). The causal hypothesis tested by SEMs assumed that LAI and leaf size are influenced by climate, that LAI is further influenced by leaf size and that all three influence primary productivity also directly. See Table S4.5 for the corresponding values.

Table S4.1 Correlation between MODIS-derived GPP and NPP with LAI and other datasets about GPP and NPP.

	LAI	GPP (MTE)	NPP (CASA)	NPP (Plot based)
**MODIS-derived GPP**	0.786	0.908	/	/
**MODIS-derived NPP**	0.733	/	0.907	0.513

Table S4.2 Coefficients of determination (R^2) for the relationships between leaf size and primary productivity among years with high or low values of primary productivity. LL, mean leaf length; LW, mean leaf width; LWs, mean leaf length-width product. See also Figure 4.5.

		Nonlinear models			Linear models		
	**Average of**	**LL**	**LW**	**LWs**	**LL**	**LW**	**LWs**
**GPP**	**2 highest years**	0.833	0.785	0.790	0.830	0.775	0.817
	**4 highest years**	0.833	0.784	0.790	0.830	0.774	0.818
	**8 highest years**	0.831	0.782	0.788	0.828	0.772	0.817
	**8 lowest years**	0.822	0.769	0.774	0.817	0.755	0.809
	**4 lowest years**	0.818	0.762	0.767	0.812	0.748	0.804
	**2 lowest years**	0.814	0.755	0.761	0.807	0.740	0.780
	**all 16 years**	0.827	0.776	0.780	0.823	0.764	0.814
**NPP**	**2 highest years**	0.803	0.762	0.779	0.792	0.742	0.796
	**4 highest years**	0.801	0.759	0.777	0.789	0.739	0.795
	**8 highest years**	0.798	0.755	0.773	0.785	0.734	0.793
	**8 lowest years**	0.780	0.728	0.749	0.763	0.703	0.778
	**4 lowest years**	0.773	0.717	0.739	0.754	0.690	0.771
	**2 lowest years**	0.767	0.708	0.730	0.747	0.680	0.764
	**all 16 years**	0.790	0.743	0.763	0.776	0.720	0.787

Table S4.3 Comparison between the effects of AET and leaf size measures on ecosystem primary productivity based on partial regressions.

R^2	MODIS-derived NPP			MODIS-derived GPP		
	LL	LW	LW.prod	LL	LW	LW.prod
**Independent effect of AET**	0.01	0.03	0.02	0.02	0.04	0.04
**Shared effect**	0.73	0.72	0.72	0.79	0.77	0.77
**Independent effect of leaf size**	0.06	0.04	0.07	0.05	0.03	0.04
**Total effect**	0.80	0.78	0.81	0.86	0.84	0.85

Table S4.4 Comparison between the effects of LAI and leaf size measures on ecosystem primary productivity based on partial regressions.

R^2	MODIS-derived NPP			MODIS-derived GPP		
	LL	LW	LW.prod	LL	LW	LW.prod
**Independent effect of LAI**	0.01	0.01	0.02	0.02	0.03	0.04
**Shared effect**	0.53	0.52	0.52	0.59	0.59	0.58
**Independent effect of leaf size**	0.25	0.20	0.27	0.23	0.18	0.23
**Total effect**	0.79	0.73	0.80	0.85	0.79	0.85

Table S4.5 The direct and indirect effect of AET on variation of primary productivity.

SEMs		Direct effect of AET	Indirect effect of AET
GPP	Length	0.36	0.55
	Width	0.46	0.45
	Length-width product	0.41	0.50
NPP	Length	0.30	0.59
	Width	0.41	0.47
	Length-width product	0.33	0.46

Note: The indirect effects are represented in the path diagrams by the following three pathways: AET → LAI → productivity, AET → leaf size → LAI → productivity, AET → leaf size → productivity. See Figure 3 for the structural equation models.

Appendix 5 **List of data sources from BIEN database used in our study, including occurrence records and range maps of all woody dicots and trait data (whole plant growth form and leaf length and width).**

[1] Enquist BJ*, et al.* Botanical big data shows that plant diversity in the New World is driven by climatic-linked differences in evolutionary rates and biotic exclusion. (Unpublished)

[2] Maitner BS*, et al.* (2018) The BIEN r package: A tool to access the Botanical Information and Ecology Network (BIEN) database. *Methods Ecol. Evol.* 9(2):373-379.

[3] Goldsmith GR*, et al.* (2016) *Plant-O-Matic*: a dynamic and mobile guide to all plants of the Americas. *Methods Ecol. Evol.* 7: 960-965. doi:10.1111/2041-210X.12548.

[4] Forest Inventory and Analysis National Program. Published online *http://www.fia.fs.fed.us/* [Accessed: 2013-1-19].

[5] Peet RK*, et al.* (2012) Vegetation-plot database of the Carolina Vegetation Survey. *Biodivers. Eco.* 4:243-253.

[6] Peet RK*, et al.* (2012) VegBank: a permanent, open-access archive for vegetation plot data. *Biodivers. Ecol.* 4:233-241.

[7] Enquist JB & Boyle B (2012) SALVIAS – the SALVIAS vegetation inventory database. *Biodivers. Ecol.* 4:288-288.

[8] Global Biodiversity Information Facility. Published online *http://www.gbif.org* [Accessed: 2012-12-11].

[9] SpeciesLink. Published online *http://www.splink.org.br* [Accessed: 2012-3-29].

Engemann K*, et al.* (2012) A plant growth form dataset for the New World. *Ecol.* 97(11): 3243-3243.

[10] Engemann K*, et al.* (2016) A plant growth form dataset for the New World. *Ecology* 97(11): 3243-3243.

[11] Kleyer M*, et al.* (2008) The LEDA Traitbase: a database of life-history traits of the Northwest European flora. *J. Ecology* 96(6): 1266-1274.

[12] Royer DL*, et al.* (2005) Correlations of climate and plant ecology to leaf size and shape: potential proxies for the fossil record. *Botanical Soc. America.* 92(7): 1141-1151.

[13] Spasojevic MJ*, et al.* (2016) When does intraspecific trait variation contribute to functional beta-diversity?. *J. Ecol.* 104: 487-496. doi:10.1111/1365-2745.12518.

[14] Price CA*, et al.* (2014) Are leaf functional traits 'invariant' with plant size and what is 'invariance' anyway?. *Funct. Ecol.* 28: 1330-1343. doi:10.1111/1365-2435.12298.

[15] Grootemaat S*, et al.* (2015) Burn or rot: leaf traits explain why flammability and decomposability are decoupled across species. *Funct. Ecol.* 29: 1486-1497. doi:10.1111/1365-2435.12449.

[16] Kraft NJB, Valencia R & Ackerly DD (2008) Functional traits and niche-based tree community assembly in an Amazonian forest. *Science* 322(5901): 580-582.

[17] Marx HE *et al.* (2016) Deconstructing DarwinA's naturalization conundrum in the San Juan Islands using community phylogenetics and functional traits. *Diversity Distrib.* 22: 318-331. doi:10.1111/ddi.12401.

Appendix 6 **Leaf size of North America and the prediction of ecosystem primary productivity.**

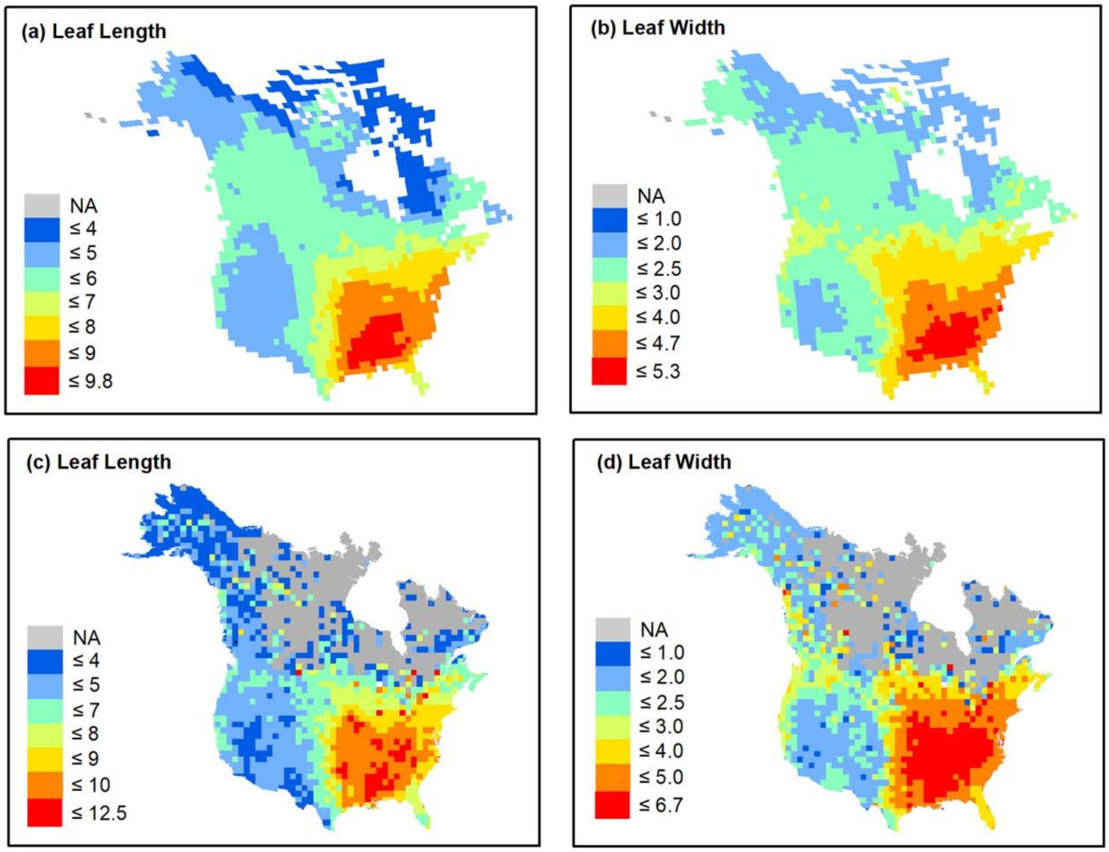

Figure S6.1 The geographic patterns in average leaf size of all woody dicots in North America. a-b) average leaf length (cm) and average leaf width (cm), respectively, based on species range maps in BIEN 2.0; c-d) average leaf length and average leaf width, respectively, based on species occurrences in BIEN 3.0. From blue to red, the values increase.

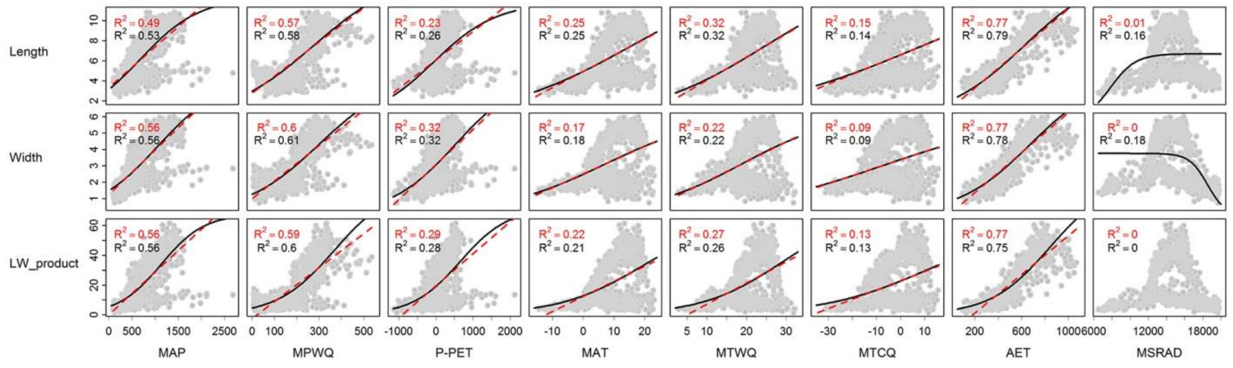

Figure S6.2 The relationships between environmental variables and mean leaf size in North America. The vertical axes represent mean leaf size (i.e. leaf length, leaf width and leaf length-width product) of woody dicots. Leaf sizes were estimated based on the distributions derived from species occurrences in the *BIEN* database. The meaning of environmental variables on the horizontal axes and lines along with text are the same as those in Figure S1.6. A modified *t*-test was applied to calculate *p* value; no curves and lines are drawn when $p > 0.05$.

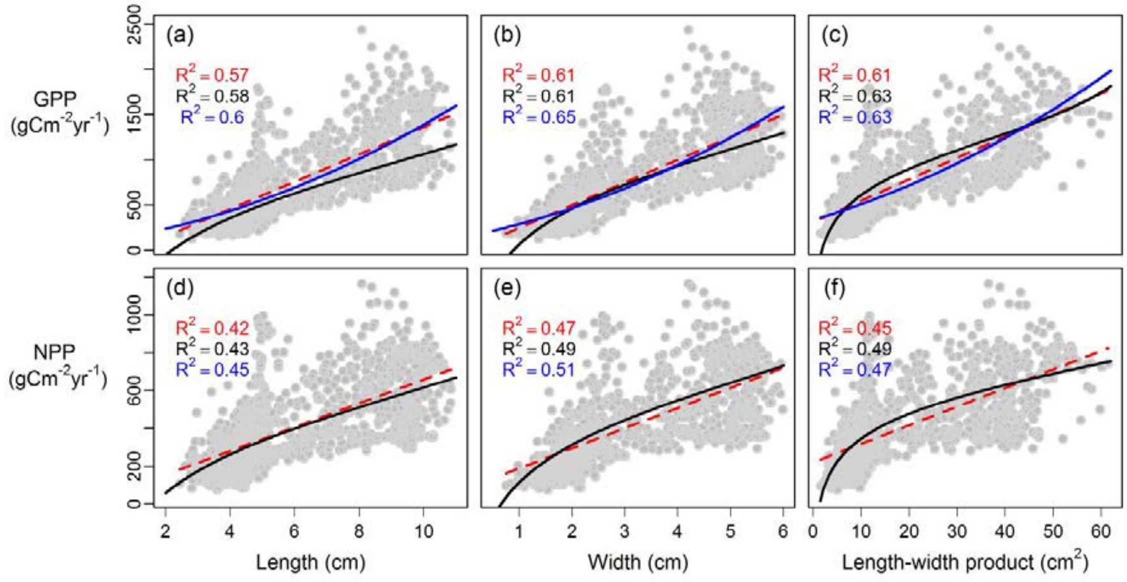

Figure S6.3 Variation in MODIS-derived primary productivity as functions of leaf size in North America. Leaf sizes were estimated based on the distributions derived from species occurrences in the *BIEN* database. The meaning of vertical axes and abscissae for the plots is the same as in Figure 2 of the main text.

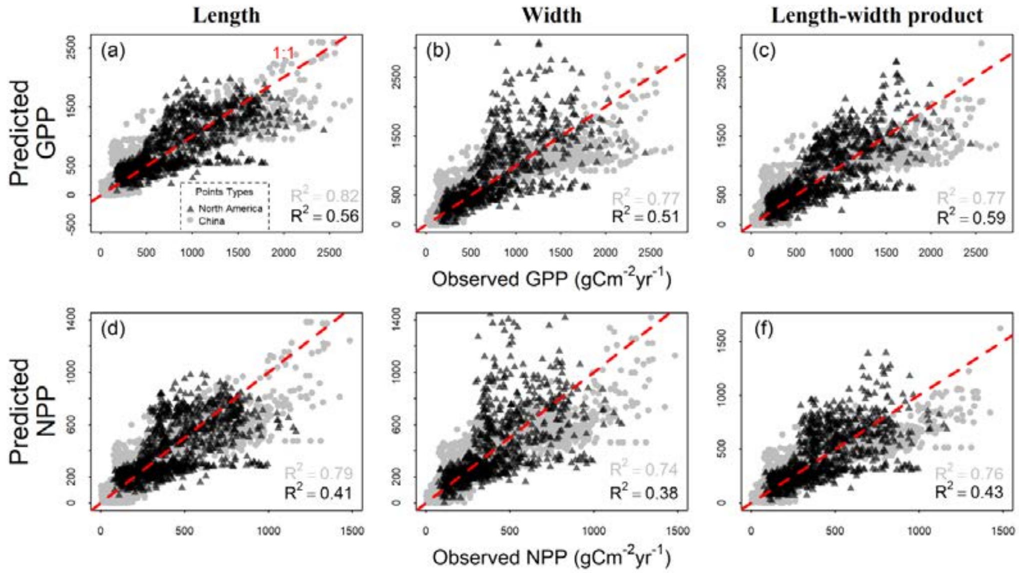

Figure S6.4 Comparison between the MODIS-derived ecosystem primary productivity and the predictions by the transfer functions of China using leaf size in in China and in North America. Leaf size in North America was estimated with species occurrences obtained from the *BIEN* database. The meaning of axes, points and legends for the plots is with the same as in Figure 4 of the main text.

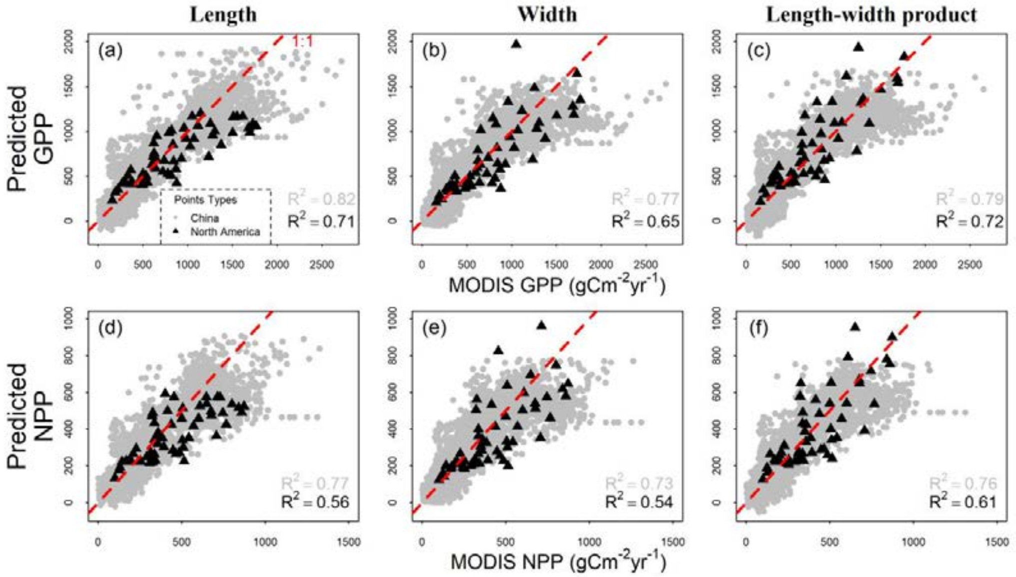

Figure S6.5 Comparison between the MODIS-derived ecosystem primary productivity and the predictions by the transfer functions of China using leaf size in in China and in North America. Leaf size in North America was estimated based on country-level distributions derived from FNA and USDA. The meaning of axes, points and legends for the plots is the same as in Figure 4 of the main text.

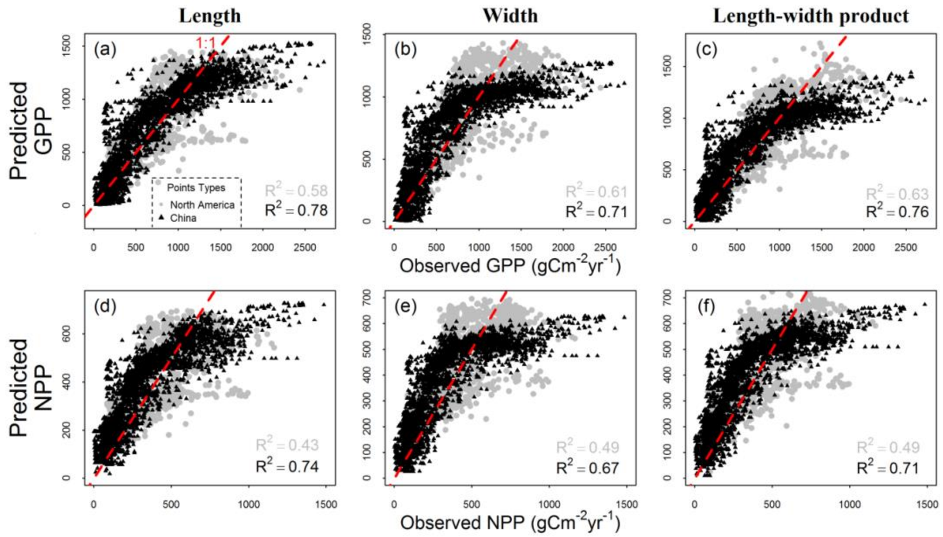

Figure S6.6 Comparison between the MODIS-derived ecosystem primary productivity and the predictions by the transfer functions of North America using leaf size in North America and in China. See Table S6.2b for the parameters. Leaf size in North America was estimated with species occurrences obtained from the *BIEN* database. The meaning of axes, points and legends for the plots is the same as in Figure 5 of the main text.

Table S6.1 Transfer functions between primary productivity and leaf size estimated with two linear models based on data from China. The transfer functions were built with data from China, and R^2 values of the best models are in bold. All relationships are significant at $p < 0.001$.

(a) Y-sqrt models: $sqrt(y) = a + bx$

y	x	a	b	c	R2	# of cells	SE	SE%
GPP	Length	3.081	13.712	15.256	0.86	3333	210.824	10.215
	Width	7.910	43.474	59.731	0.84	3332	240.671	13.559
	Length-width product	109.015	17.091	0.670	0.82	3332	241.068	9.457
NPP	Length	6.720	13.413	6.693	0.82	3333	118.701	12.150
	Width	10.953	33.843	26.143	0.80	3332	131.816	15.635
	Length-width product	68.269	9.044	0.300	0.80	3332	122.563	10.139

(b) Linear models: $y = a + bx$

y	*x*	*a*	*b*	R^2	# of cells	SE	SE%
GPP	Length	-400.402	191.430	0.82	3333	223.678	8.238
	Width	-322.875	369.259	0.76	3332	258.382	9.516
	Length-width product	17.103	27.082	0.81	3332	229.679	8.459
NPP	Length	-168.073	91.392	0.77	3333	123.839	8.345
	Width	-130.992	176.266	0.72	3332	138.432	9.328
	Length-width product	27.161	13.098	0.79	3332	120.728	8.135

Note: In (a), *a, b* and *c* are the coefficients of the quadratic model $y=a+bx+cx^2$. We first conducted y-sqrt-transformed models as $\sqrt{y} = a + b \times x$ to extract the coefficients considering the distribution of residuals and goodness of fits for the regressions, and then transformed the models as $y = a + bx + cx^2$ to predict *y*. In (b), *a* and *b* are the intercept and slope of linear regressions. See Table 1 for more information about the variables.

Table S6.2 Transfer functions between ecosystem primary productivity and leaf size estimated with the nonlinear models, $y = \frac{1}{r}(\alpha\text{-}\ln(\frac{K}{x} - 1))$, $x < K$, based on data from North America, (a) using distribution derived from range maps; (b) using distribution derived from occurrence records. GPP and NPP used here were derived from MODIS product. All relationships were significant at $p < 0.001$.

(a) Non-linear transfer functions based on range maps

y	*x*	***K***	***a***	***r***	**R^2**	**# of cells**	**SE**	**SE%**
**GPP**	*Length*	16.320	1.448	0.001	0.65	1564	243.537	10.968
	Width	8.848	1.816	0.001	0.67	1537	235.710	10.624
	Length-width product	95.531	3.249	0.002	0.68	1529	231.756	10.607
**NPP**	*Length*	16.320	1.717	0.003	0.49	1564	141.309	12.428
	Width	5.506	0.981	0.002	0.47	1537	225.450	19.88
	Length-width product	95.531	3.728	0.005	0.51	1529	139.820	12.533

(b) Non-linear transfer functions based on occurrence records

y	*x*	***K***	***a***	***r***	**R^2**	**# of cells**	**SE**	**SE%**
**GPP**	*Length*	19.234	2.051	0.002	0.58	955	299.674	12.557
	Width	11.232	2.458	0.002	0.61	931	287.309	12.039
	Length-width product	72.383	3.664	0.003	0.63	924	280.898	12.131
**NPP**	*Length*	19.234	2.386	0.004	0.43	955	167.786	14.833
	Width	11.232	2.784	0.004	0.49	931	159.951	14.141
	Length-width product	121.267	4.485	0.006	0.49	924	159.940	14.674

Table S6.3 Transfer functions between ecosystem primary productivity and leaf size estimated with the linear models, $y = a + bx$, based on data from North America, (a) using distribution derived from range maps; (b) using distribution derived from occurrence records. GPP and NPP used here were derived from MODIS product. All relationships were significant at $p < 0.001$.

(a) Linear transfer functions based on range maps

y	*x*	***a***	***b***	**R^2**	**# of cells**	**SE**	**SE%**
**GPP**	*Length*	-591.741	224.882	0.65	1546	243.676	16.522
	Width	-295.810	369.746	0.66	1537	238.741	17.729
	Length-width product	222.693	21.843	0.65	1529	244.831	16.471
**NPP**	*Length*	-149.257	23.032	0.49	1564	142.257	23.032
	Width	-21.534	153.500	0.49	1537	142.337	25.46
	Length-width product	198.302	8.877	0.46	1529	146.767	24.30

(b) Linear transfer functions based on occurrence records

y	*x*	***a***	***b***	**R^2**	**# of cells**	**SE**	**SE%**
**GPP**	*Length*	-149.832	151.414	0.58	955	300.365	23.631
	Width	-1.982	250.790	0.61	931	290.578	22.099
	Length-width product	310.443	15.819	0.61	924	288.640	20.327
**NPP**	*Length*	28.784	62.917	0.42	955	169.088	32.014
	Width	28.784	62.917	0.42	955	169.088	32.014
	Length-width product	220.107	6.550	0.45	924	166.384	28.297

Table S6.4 Summary of obtained leaf fossils.

Sites	Geologic time	# of species with fossils	Mean leaf length (cm)	Mean leaf width (cm)
Fushun, Liaoning	Eocene	52	6.43	3.44
Jinggu, Yunnan	Oligocene	25	6.49	2.67
Linqu, Shandong	Miocene	136	8.27	4.62
Ninghai, Zhejiang	Miocene	16	6.04	3.49
Xiaolongtan, Yunnan	Late Miocene to early Pliocene	15	6.18	3.11

Note: To further demonstrate the usage of leaf size for the reconstruction of paleo-primary productivity, we collected all available atlas of palaeontology and ancient plants found in China to extract the leaf size of fossil leaves. In total, we obtained 417 sets of species-by-site leaf size from fossils.

Table S6.5 Reconstruction of paleo-climate and paleo-primary productivity from leaf fossils. The reconstruction was based on the transfer functions built using Chinese data.

Sites	MAT_{pred} (°C)	MAP_{pred} (mm)	AET_{pred} (mm)	GPP_{pred} (gC $m^{-2}yr^{-1}$)	NPP_{pred} (gC $m^{-2}yr^{-1}$)
Fushun, Liaoning province	9.96	781.34	616.07	814.62	410.64
Jinggu, Yunnan province	8.67	665.37	539.19	696.05	355.02
Linqu, Shandong province	14.80	1126.64	949.91	1133.92	559.69
Ninghai, Zhejiang Province	9.72	761.23	602.92	793.77	400.73
Xiaolongtan, Yunnan province	9.15	704.11	567.61	737.03	374.40

References

Boyle, B., Hopkins, N., Lu, Z., Garay, J.A.R., Mozzherin, D., Rees, T. *et al.* (2013). The taxonomic name resolution service: an online tool for automated standardization of plant names. *BMC Bioinformatics*, 14.

Blomberg, S.P., Garland, T. & Ives, A.R. (2003). Testing for phylogenetic signal in comparative data: Behavioral traits are more labile. Evolution, 57, 717-745.

Bruelheide, H., Dengler, J., Purschke, O., Lenoir, J., Jiménez-Alfaro, B., Hennekens, S.M. *et al.* (2018). Global trait–environment relationships of plant communities. *Nature Ecology & Evolution*, 2, 1906-1917.

Cain, S.A. & Castro, G.d. (1959). Manual of vegetation analysis. *Science*, 131, 1218-1219.

Cristofori, V., Rouphael, Y., Gyves, E.M.-d. & Bignami, C. (2007). A simple model for estimating leaf area of hazelnut from linear measurements. *Sci. Hortic.*, 113, 221-225.

Fang, J., Wang, Z. & Tang, Z. (2011). *Atlas of woody plants in China: distribution and climate*. Springer.

Feng, Y., Zhu, J., Zhao, X., Tang, Z., Zhu, J. & Fang, J. (2019). Changes in the trends of vegetation net primary productivity in China between 1982 and 2015. *Environmental Research Letters*, 14, 124009.

Garbulsky, M.F., Peñuelas, J., Papale, D., Ardö, J., Goulden, M.L., Kiely, G. *et al.* (2010). Patterns and controls of the variability of radiation use efficiency and primary productivity across terrestrial ecosystems. *Global Ecol. Biogeogr.*, 19, 253-267.

Goldsmith, G.R., Morueta-Holme, N., Sandel, B., Fitz, E.D., Fitz, S.D., Boyle, B. *et al.* (2016). Plant-O-Matic: a dynamic and mobile guide to all plants of the Americas. *Methods Ecol. Evol.*, 7, 960-965.

Hengl, T., Mendes de Jesus, J., Heuvelink, G.B.M., Ruiperez Gonzalez, M., Kilibarda, M., Blagotić, A. *et al.* (2017). SoilGrids250m: Global gridded soil information based on machine learning. *PLoS One*, 12, e0169748.

Hijmans, R.J., Cameron, S.E., Parra, J.L., Jones, P.G. & Jarvis, A. (2005). Very high resolution interpolated climate surfaces for global land areas. *Int. J. Climatol.*, 25, 1965-1978.

Klein, T., Randin, C. & Körner, C. (2015). Water availability predicts forest canopy height at the global scale. *Ecol. Lett.*, 18, 1311-1320.

Lieth, H. (1975). Modeling the primary productivity of the world. Primary productivity of the biosphere (ed. by H. Lieth and R.H. Whittaker). *Springer, New York.*, 237-263.

Liu, Y., Liu, R. & Chen, J.M. (2012). Retrospective retrieval of long-term consistent global leaf area index (1981-2011) from combined AVHRR and MODIS data. *J. Geophys. Res. Biogeosci.*, 117.

Lu, L.-M., Mao, L.-F., Yang, T., Ye, J.-F., Liu, B., Li, H.-L. *et al*. (2018). Evolutionary history of the angiosperm flora of China. Nature, 554, 234-238.

Magallon, S., Gomez-Acevedo, S., Sanchez-Reyes, L.L. & Hernandez-Hernandez, T. (2015). A metacalibrated time-tree documents the early rise of flowering plant phylogenetic diversity. New Phytol., 207, 437-453.

Maire, V., Wright, I.J., Prentice, I.C., Batjes, N.H., Bhaskar, R., van Bodegom, P.M. *et al.* (2015). Global effects of soil and climate on leaf photosynthetic traits and rates. *Global Ecol. Biogeogr.*, 24, 706-717.

Maitner, B.S., Boyle, B., Casler, N., Condit, R., Donoghue, J., Durán, S.M. *et al.* (2018). The bien r package: A tool to access the Botanical Information and Ecology Network (BIEN) database. *Methods Ecol. Evol.*, 9, 373-379.

Michaletz, S.T., Cheng, D., Kerkhoff, A.J. & Enquist, B.J. (2014). Convergence of terrestrial plant production across global climate gradients. *Nature*, 512, 39-43.

Peppe, D.J., Royer, D.L., Cariglino, B., Oliver, S.Y., Newman, S., Leight, E. *et al.* (2011). Sensitivity of leaf size and shape to climate: global patterns and paleoclimatic applications. *New Phytol.*, 190, 724-739.

Piedallu, C., Gegout, J.C., Perez, V. & Lebourgeois, F. (2013). Soil water balance performs better than climatic water variables in tree species distribution modelling. *Global Ecol. Biogeogr.*, 22, 470-482.

Rouphael, Y., Mouneimne, A.H., Ismail, A., Mendoza-De Gyves, E., Rivera, C.M. & Colla, G. (2010). Modeling individual leaf area of rose (Rosa hybrida L.) based on leaf length and width measurement. *Photosynthetica*, 48, 9-15.

Traiser, C., Klotz, S., Uhl, D. & Mosbrugger, V. (2005). Environmental signals from leaves - a physiognomic analysis of European vegetation. *New Phytol.*, 166, 465-484.

United Nations Environment, P. (1992). *World atlas of desertification / UNEP, United Nations Environment Programme*. Edward Arnold, London ; Baltimore.

Wang, Z., Fang, J., Tang, Z. & Lin, X. (2011). Patterns, determinants and models of woody plant diversity in China. *Proc. R. Soc. B*, 278, 2122-2132.

Wang, Z., Li, Y., Su, X., Tao, S., Feng, X., Wang, Q. *et al.* (2019). Patterns and ecological determinants of woody plant height in eastern Eurasia and its relation to primary productivity. *J Plant Ecol*.

Wilf, P., Wing, S.L., Greenwood, D.R. & Greenwood, C.L. (1998). Using fossil leaves as paleoprecipitation indicators: An Eocene example. *Geology*, 26, 203-206.

Yao, Y., Wang, X., Li, Y., Wang, T., Shen, M., Du, M. *et al.* (2018). Spatiotemporal pattern of gross primary productivity and its covariation with climate in China over the last thirty years. *Global Change Biol.*, 24, 184-196.

Yuan, W., Liu, S., Yu, G., Bonnefond, J.-M., Chen, J., Davis, K. *et al.* (2010). Global estimates of evapotranspiration and gross primary production based on MODIS and global meteorology data. *Remote Sens. Environ.*, 114, 1416-1431.

Zanne, A.E., Tank, D.C., Cornwell, W.K., Eastman, J.M., Smith, S.A., FitzJohn, R.G. *et al.* (2014). Three keys to the radiation of angiosperms into freezing environments. Nature, 506, 89-92.
